# Improving pediatric COVID-19 vaccine uptake using an mHealth tool (MoVeUp): study protocol for a randomized, controlled trial

**DOI:** 10.1186/s13063-022-06819-3

**Published:** 2022-10-28

**Authors:** Russell J. McCulloh, Paul M. Darden, Jessica Snowden, Songthip Ounpraseuth, Jeannette Lee, Martina Clarke, Sophia R. Newcomer, Linda Fu, DeAnn Hubberd, Jaime Baldner, Maryam Garza, Ellen Kerns

**Affiliations:** 1 Children’s Hospital & Medical Center, 8200 Dodge St., Omaha, NE 68114 USA; 2 University of Arkansas for Medical Sciences, 4301 West Markham, Little Rock, AR 72205 USA; 3 College of Information Science & Technology, University of Nebraska Omaha, 172 Peter Kiewit Institute, 1110 South 67th Street, Omaha, NE 68182 USA; 4 School of Public Health and Community Health Sciences, University of Montana, Skaggs Building Room 177, 32 Campus Drive, Missoula, MT 59812 USA; 5 Office of the Director, National Institutes of Health, 11601 Landsdown Sreet, Rockville, MD 20852 USA; 6 University of Nebraska Medical Center, 42nd and Emile St., Omaha, NE 68131 USA

**Keywords:** Vaccination, Vaccine hesitancy, Vaccine confidence, mHealth, Mobile health, COVID-19, SARS-CoV-2

## Abstract

**Background:**

Coronavirus disease 2019 (COVID-19) vaccines demonstrate excellent effectiveness against infection, severe disease, and death. However, pediatric COVID-19 vaccination rates lag among individuals from rural and other medically underserved communities. The research objective of the current protocol is to determine the effectiveness of a vaccine communication mobile health (mHealth) application (app) on parental decisions to vaccinate their children against COVID-19.

**Methods:**

Custodial parents/caregivers with ≥ 1 child eligible for COVID-19 vaccination who have not yet received the vaccine will be randomized to download one of two mHealth apps. The intervention app will address logistical and motivational barriers to pediatric COVID-19 vaccination. Participants will receive eight weekly push notifications followed by two monthly push notifications (cues to action) regarding vaccinating their child. Through branching logic, users will access customized content based on their locality, degree of rurality-urbanicity, primary language (English/Spanish), race/ethnicity, and child’s age to address COVID-19 vaccine knowledge and confidence gaps. The control app will provide push notifications and information on general pediatric health and infection prevention and mitigation strategies based on recommendations from the American Academy of Pediatrics (AAP) and the Centers for Disease Control and Prevention (CDC). The primary outcome is the proportion of children who complete COVID-19 vaccination series. Secondary outcomes include the proportion of children who receive ≥ 1 dose of COVID-19 vaccine and changes in parent/caregiver scores from baseline to immediately post-intervention on the modified WHO SAGE Vaccine Hesitancy Scale adapted for the COVID-19 vaccine.

**Discussion:**

The COVID-19 pandemic inflicts disproportionate harm on individuals from underserved communities, including those in rural settings. Maximizing vaccine uptake in these communities will decrease infection rates, severe illness, and death. Given that most US families from these communities use smart phones, mHealth interventions hold the promise of broad uptake. Bundling multiple mHealth vaccine uptake interventions into a single app may maximize the impact of deploying such a tool to increase COVID-19 vaccination. The new knowledge to be gained from this study will directly inform future efforts to increase COVID-19 vaccination rates across diverse settings and provide an evidentiary base for app-based vaccine communication tools that can be adapted to future vaccine-deployment efforts.

**Clinical trials registration:**

ClinicalTrials.gov NCT05386355. Registered on May 23, 2022.

## Background

Coronavirus disease 2019 (COVID-19) vaccines demonstrate excellent effectiveness (50–90%) against infection and symptomatic disease, and more importantly, almost 100% protection from severe disease and death [1, 2]. However, nearly 3 in 10 Americans state they probably or definitely will not get vaccinated, particularly individuals from rural, low-income, or medically underserved communities [3]. Moreover, individuals from Black and Hispanic communities express higher vaccine hesitancy, despite experiencing significantly worse disease burden and mortality [4–7]. The National Institutes of Health (NIH) has identified these vulnerable populations, as high priorities for vaccination and vaccine communication efforts [8]. Key goals of vaccine communication highlighted by the NIH include (1) providing assurance of vaccine safety; (2) highlighting collective and individual benefits to vaccination; (3) explaining the vaccine development and approval process; (4) addressing vaccine hesitancy; and (5) monitoring and countering misinformation. These communication objectives require tailoring to specific target audiences to be effective and must, therefore (1) leverage close community partnerships to identify relevant information needs and cultural context and (2) employ scalable and impactful communication strategies that encourage vaccination across diverse segments of the population [9].

Currently, the Moderna, Pfizer BioNTech COVID-19, and Novovax vaccines are authorized by the US Food and Drug Administration (FDA) for use in children in ages 6 months and older, which includes 2- and 3-dose series for some age groups and vaccine formulations [10]. Severe acute respiratory syndrome coronavirus 2 (SARS-CoV-2), the virus that causes COVID-19 disease, has infected more than 3 million children and killed more than 1000 children since 2020. High COVID-19 vaccination coverage among children is critical to lessen the public health burden on children’s physical, mental, and social health [11, 12].

### Parents/caregivers vary in their confidence/hesitancy to vaccinate their children against SARS-CoV-2

While there is a good amount of literature on adults and vaccine hesitancy, there is much less information about children. A recent nationally representative study of parents’ intentions and perceptions of COVID-19 vaccination was published by Szilagyi et al. [13] The survey covered February 17 to March 30, 2021. In that survey, a parent’s race and ethnicity were associated with the likelihood of their child getting vaccinated with 44% of parents who were White and 47% of parents who were Black answering somewhat or very unlikely to vaccinate their child versus 42% overall and 36% of parents who were Hispanic and 35% of Asian American. Age of parent and of their child were associated with younger ages being more likely to answer somewhat or very unlikely to vaccinate their child. The most trusted source of information about the COVID-19 vaccine was the child’s doctor, with 72% of parents indicating that they completely or mostly trust their child’s doctor. Interestingly, 50% indicated trust in the CDC and 48% in the AAP. No other source of information approached 50%. When these results were analyzed controlling for parent and child sociodemographic factors, parent perceptions, child receipt of influenza vaccine, and parental receipt of COVID-19 vaccine, trust in the child’s doctor remained significant while other parent and child factors, including child’s age and race/ethnicity, were no longer significant predictors of indicating very likely or likely to get a vaccine for their child. It is worth noting that, when surveyed, American adults’ intent to vaccinate has changed and gotten more positive over time [13, 14].

### Vaccine hesitancy is higher in rural communities and ISPCTN states, which are an ideal setting for testing the effectiveness of an mHealth Vaccine Uptake app

The Environmental influences on Child Health Outcomes IDeA States Pediatric Clinical Trials Network (ECHO ISPCTN) includes 18 states from geographically diverse areas of the USA [15]. Unfortunately, most ISPCTN states report lower than average rates of COVID-19 vaccine uptake. Many ECHO ISPCTN states have a disproportionately high number of rural residents compared with non-ISPCTN states, and COVID-19 vaccine hesitancy—and vaccine coverage rates—lags significantly in rural communities across the US when compared to their non-rural counterparts [16]; only 52% of rural Americans ≥ 5 years old have completed their primary COVID-19 vaccine series versus 66% of urban Americans. Community feedback from ECHO ISPCTN sites highlights significant vaccine hesitancy and barriers to COVID-19 vaccination. For example, community advisory board feedback from stakeholders in rural Nebraska (one of the ECHO ISPCTN states) emphasize that access to COVID-19 vaccine among eligible children and their families, particularly those with low English fluency, is low. Additionally, there is significant concern from community members regarding the safety of COVID-19 vaccines. In Mississippi, surveys of rural families across diverse racial and ethnic backgrounds demonstrate very high levels of COVID-19 vaccine hesitancy, and in some higher-risk groups such as rural Black families, 40% or more of respondents may be vaccine hesitant. Hesitancy among rural Black individuals in Mississippi aligns with findings of lower vaccine uptake nationally. Overall, 47% of Black Americans are vaccinated against COVID-19 versus 52% of White Americans [17].

### Guiding conceptual framework: The Health Belief Model

**Fig. 1:**
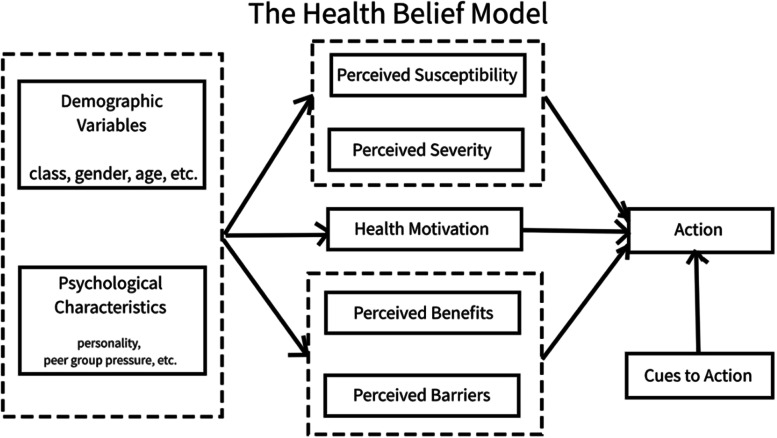
Health Belief Model components

Many factors contribute to an individual’s decision to engage in a health promotion or disease prevention activity. The Health Belief Model (HBM), first developed in the early 1950s, provides an organizing framework to understand people’s health behavior decisions [18]. The HBM relies on the assumption that individuals’ health decisions stem from a desire to avoid and/or recover from illness and an individual’s belief that they can take specific actions to prevent, cure, or reduce the severity of illness. The HBM has been used to assess vaccine hesitancy for other vaccines, including influenza and human papillomavirus (HPV) vaccines [19, 20]. Specific components of the HBM are summarized in Fig. 1 [21].

The HBM provides an organizational framework for understanding the considerations of parents and caregivers in deciding whether to vaccinate their children against COVID-19. HBM constructs as applied to parental COVID-19 vaccine decision-making include:

1. Perceived susceptibility of their children being infected with SARS-CoV-2
2. Perceived severity of COVID-19 disease or multisystem inflammatory syndrome in children (MIS-C) in their children.
3. Perceived benefits that their children may experience from receiving a COVID-19 vaccine.
4. Perceived barriers (both attitudinal and logistic) that parents/caregivers may have to obtaining COVID-19 vaccination for their children
5. Cues to action (personal stories/vignettes, advice, and prompts received from others, including social norms) to trigger a parent/caregiver to vaccinate their children against COVID-19
6. Self-efficacy or belief in one’s own capacity to complete the steps necessary to vaccinate their children against COVID-19 (e.g., identifying a vaccination site, scheduling an appointment, arranging transportation, being able to pay)

Other level factors may influence the above elements, including race, ethnicity, and education level, among others. mHealth vaccine communication apps can address multiple domains of the Health Belief Model simultaneously and hold the potential for customization to incorporate relevant community, cultural, and/or individual factors [22], which should improve the effectiveness of the app to increase COVID-19 vaccination rates among app users.

### mHealth tools can increase vaccine confidence and vaccination rates

mHealth tools, such as mobile apps and decision support tools, demonstrate the potential to positively affect vaccination rates [23–26]. In an Italian study of a smartphone app to increase parents’ knowledge and empowerment when deciding to vaccinate their child against measles, mumps, and rubella (MMR), use of the smartphone app increased parental knowledge and confidence in choosing MMR vaccination and increased parental-reported intention to vaccinate. However, actual immunization actions taken after the intervention period were not measured. Similarly, in a cluster-randomized trial of a web-based decision aid for informing parental decision-making about MMR vaccination, the web-based tool, when compared to a leaflet intervention, was associated with lower parental-reported decisional conflict and higher vaccination rates among parents deciding whether to give the first dose of MMR vaccine to their child. A follow-up study of the web-based tool also found it more cost-effective than traditional approaches [26].

*Cues to action*, which can include behavioral nudges, are a proven effective means of increasing vaccine uptake in various settings [27, 28]. Text messages and voice phone calls can serve as effective prompts to action, and such prompts maximize their impact when they include customized information relevant to the individual recipient. In a large field experiment conducted in the USA, text message alerts alone increased flu vaccination rates among 49,000 participants by 5%. Nudges were most effective when they were phrased as there being a vaccine reserved for a participant’s child and when they used language that would be expected to come from their healthcare provider.

*Narrative messaging and vignettes*can also serve as prompts to action. Narrative messaging can present information to users in a culturally congruent context and has been shown to increase vaccination rates, particularly in HPV vaccination [29, 30]. A systematic review of educational interventions for increasing HPV vaccination identified two interventional trials that evaluated the impact of narrative stories on vaccination outcomes at up to 2 months. In one study, adolescent participants who viewed narrative videos featuring peers and experts were twice as likely to be vaccinated as those viewing other message formats [31]. One example of using narrative interventions to promote vaccine uptake is the CANImmunize app, originally piloted as ImmunizeCA [24, 32]. The CANImmunize app, used in Canada to assist in tracking of vaccination status and to deliver information on vaccines to users, includes video education and personal endorsements for vaccines, combined with push notifications, medical records keeping, and self-directed learning to increase vaccination uptake. Initial reports suggest high usage of the tool, including narrative elements, but controlled trials of the CANImmunize app on vaccination rates are lacking.

*Tailored messaging*, *including culturally congruent storytelling*, can provide information to users in a less-threatening and more relatable format by using likable messengers with similarities to the end-user. Such messaging can engender an emotional connection, which may help persuade individuals to engage in recommended health behaviors [33].

In summary, prior research highlights the potential impact of several mHealth interventions to increase vaccine acceptance and uptake. However, findings from these studies may not be directly applicable to COVID-19 vaccination. Prior mHealth-based interventional and large-scale clinical trials assessing the effectiveness of mHealth vaccine uptake tools are generally lacking. Given that national efforts to use mHealth tools to increase vaccine uptake have been deployed in other countries [32], establishing an evidentiary base for their effectiveness may have major public health program development and implementation implications. Additionally, prior interventions focus on vaccines that had been licensed through the standard FDA approval process following 10 to 15 years of research and development. By contrast, COVID-19 vaccines are only available to some US age groups under FDA EUA to facilitate the availability of unapproved medical treatments during public health emergencies. EUA for the first COVID-19 vaccines came after an unprecedented 1 year of development and testing. Multiple surveys demonstrate that the newness of these vaccines raises safety concerns for many individuals [34]. Finally, many mHealth interventions lack customization for specific community- and/or individual-level factors that may influence vaccine decision-making, such as race, ethnicity, or geography (such as rurality). Our mHealth Vaccine Uptake app uniquely combines several proven effective methods for increasing vaccination rates with user customization intended to increase pediatric COVID-19 vaccination rates across diverse settings.

### Evidence of capability: the Children’s COVID-19 Student Symptom Checker and mHealth software platform

**Fig. 2:**
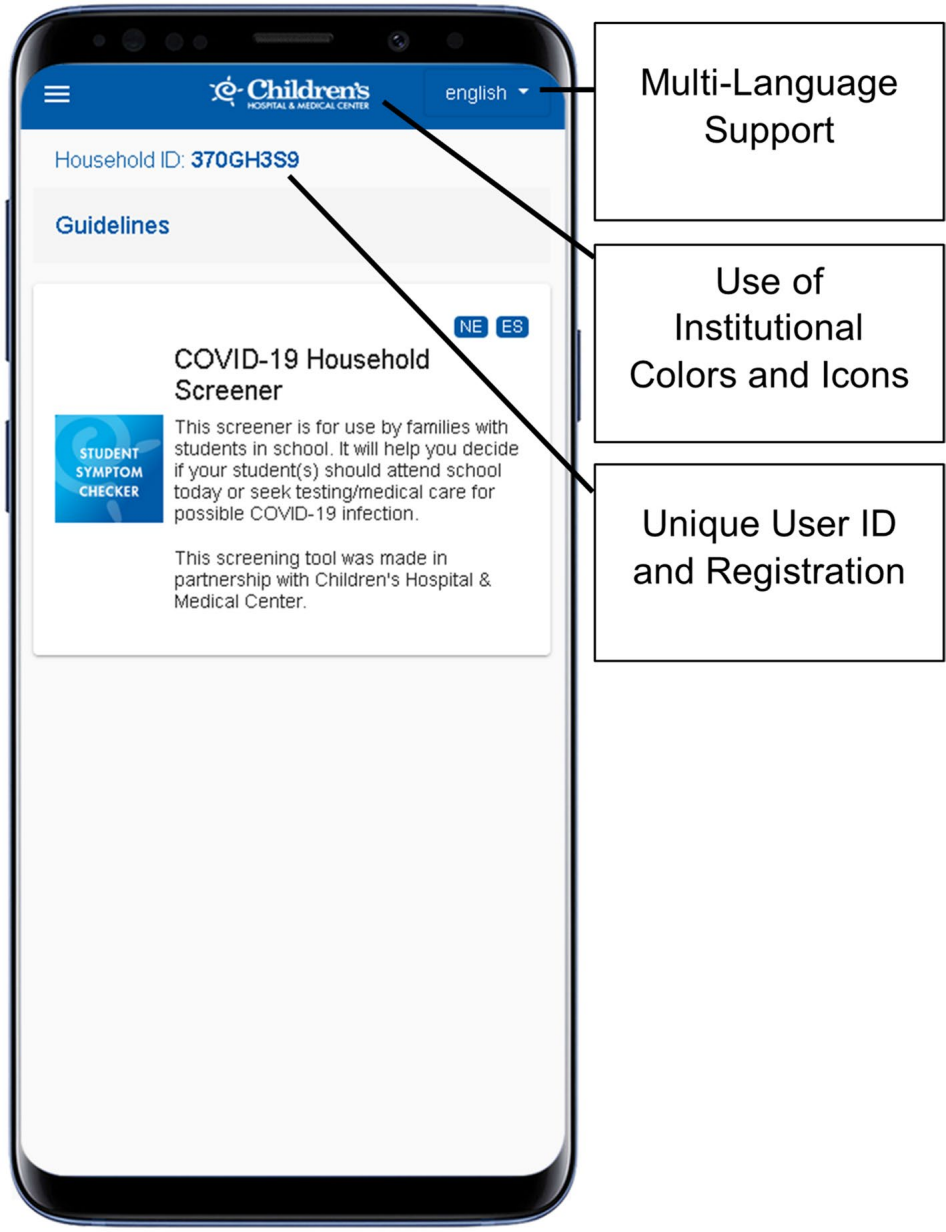
Screenshot of the Children’s COVID-19 Student Symptom Checker

The co-PIs of this protocol have extensive experience developing and deploying customizable mHealth clinical decision support apps for healthcare providers. These apps have been used by > 50,000 users and incorporate user analytics that enable assessment of engagement with the app [35–37]. We have also created customizable mHealth apps for COVID-19 infection response decision-making for parents (COVID-19 Student Symptom Checker) that, in partnership with community stakeholders and school districts, have been translated into multiple languages, including Spanish and Nepali (Fig. 2).

These apps were built using software and data infrastructure developed at and owned by the University of Nebraska Medical Center (UNMC). The important functionalities of the previous two apps that will also be deployed for the Vaccine Uptake app in the current protocol are:

1. Customization at both the local institution and individual user levels for an experience that is uniquely tailored to each user’s local context and personal background.
2. A development environment that enables content viewing in multiple languages.
3. A content management interface that enables rapid content modification and updates.
4. Push notifications to participants to trigger app usage.
5. Back-end analytics to measure user interactions with app components.

**Fig. 3:**
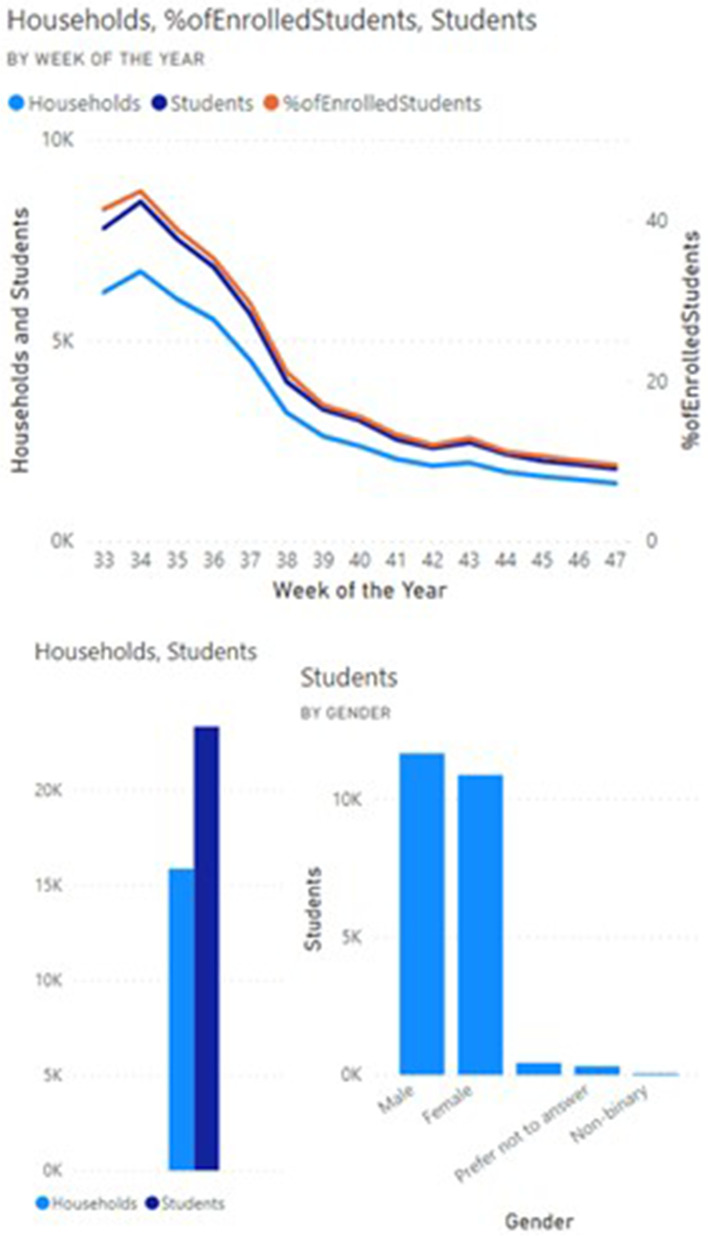
Sample dashboard of enrollment metrics generated from data provided by users of the Student Symptom Checker

**Fig. 4:**
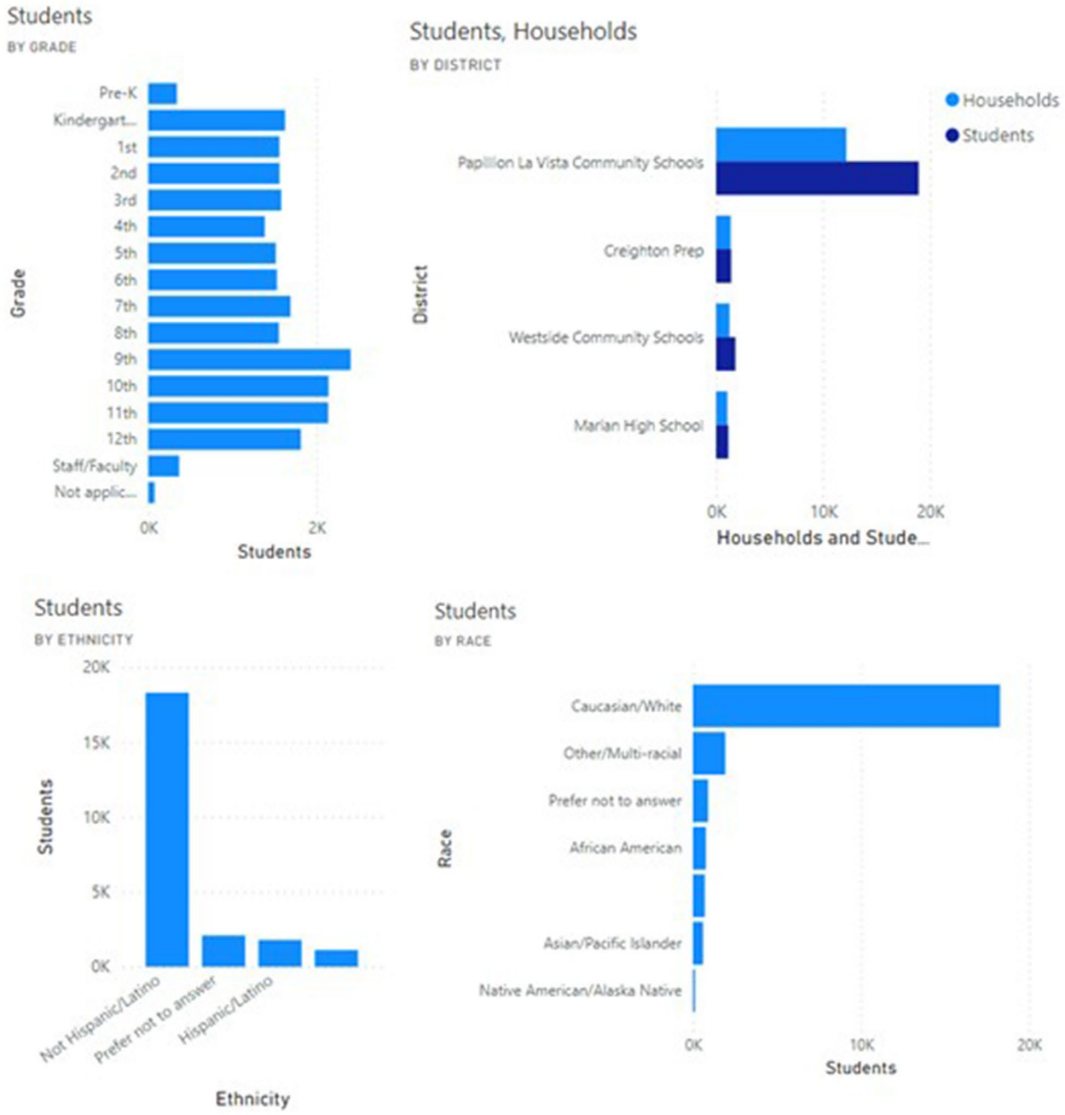
Sample dashboard of enrollment metrics generated from data provided by users of the Student Symptom Checker

Data since release of the COVID-19 Student Symptom Checker app in August 2020 shows that these tools demonstrate broad uptake and use across diverse racial and ethnic backgrounds, with more than 85% of eligible households having registered for the tool. The median weekly number of students screened by the app was 7000 (Figs. 3 and 4). User feedback demonstrated that parents used the app to make decisions on whether to obtain SARS-CoV-2 testing and whether to send their children to school. Feedback from school districts showed that school nurses and principals used the app to help them counsel parents regarding school attendance and to review components of school response plans included within the app.

Finally, members of our team have experience developing and testing the effectiveness of vaccine communication interventions. Specifically, Dr. Paul Darden has assessed the impact of text messaging interventions on HPV vaccination rates [38]; conducted prospective clinical trials of multi-modal, clinic-based interventions to increase HPV vaccination rates [39]; and assessed parental perceptions of barriers to vaccination through the Pediatric Research in Outpatient Settings (PROS) Network, a national practice-based research network [40].

## Methods/design

### Overall design

This multisite, parallel, randomized, controlled trial will assign parents/caregivers of children eligible to receive COVID-19 vaccine seen at participating clinics to receive either the mHealth Vaccine Uptake app or the General Health app containing general infection and child health topics. Randomization will be stratified by site using a permuted block design with varying block size. Outcome is receipt of COVID-19 vaccine by the parents’/caregivers’ children.

**Table:** 

Objectives	Endpoints	Justification for endpoints
Primary		
Objective 1: Determine the effect of a parent-facing, vaccination decision-making mHealth tool on children’s COVID-19 vaccine series completion	Proportion of children who complete COVID-19 vaccination, as verified in state, clinic, or participant-held records	The intended goal of the intervention is to increase the number of children receiving a complete COVID-19 vaccine series since series completion provides children with maximal protection afforded by vaccination. Self-report of vaccination will not be used, only verified vaccination records, to avoid introduction of recall and social desirability biases in the assessment of the endpoint. The study team will define vaccine series completion as per the current Advisory Committee on Immunization Practices (ACIP) guidance for the vaccine product. For children who receive a product that requires more than 2 doses for the primary series, receipt of up to 3 doses will be considered complete
Secondary		
Objective 1: Determine the effect of a parent-facing, vaccination decision-making mHealth tool on children’s COVID-19 vaccine series initiation	Proportion of children who receive ≥ 1 dose of the COVID-19 vaccination series, as verified in state, clinic, or participant-held records	Some COVID-19 vaccines require completion of a multiple-dose series to achieve intended immunity. A necessary precursor to our primary endpoint of vaccine series completion is vaccine series initiation (i.e., receipt of first dose). As more individuals initiate vaccine series than complete it, vaccine series initiation may be a more sensitive indicator of the intervention’s effect
Objective 2: Determine the effect of a parent-facing, vaccination decision-making mHealth tool on parental attitude toward pediatric COVID-19 vaccination	Change in enrolled parent/caregiver domain scores from baseline to week 16 on the modified SAGE Vaccine Hesitancy Scale adapted for the COVID-19 Vaccine	Vaccine hesitancy contributes to vaccination decisions. Measuring any changes in vaccine hesitancy in association with use of the Vaccine Uptake app will provide further insight into the impact of the app on parent/caregiver decision-making

#### Rationale for a randomized trial

Based on the Health Belief Model and prior evidence, factors contributing to individuals’ vaccine hesitancy, confidence, and/or refusal likely are multi-factorial and vary widely across individuals. Randomization of participants to the Vaccine Uptake or the General Health app study arms, outlined further below, is designed to minimize selection bias of individuals with specific attitudes or beliefs into a single study arm. For example, individuals who have lower vaccine hesitancy may be at baseline more interested/open to participating in the Vaccine Uptake study arm, whereas individuals with greater hesitancy may be more likely to decline [41]. This potential bias ideally will be minimized by having participants consent to engaging in whatever study arm to which they are randomly assigned.

#### Rationale for using the mHealth app platform developed at UNMC in the intervention and control study arms

Most parents/caregivers, including those in rural areas, have access to a mobile device and the internet [42]. Prior studies support the feasibility of deploying app-based interventions aimed at increasing vaccination rates. Based on community feedback and our team’s experience deploying app-based interventions for COVID-19 symptom screening, ensuring that the app imposes a low data burden is important to maximizing uptake. Consequently, the platform developed at the UNMC uses data at only 4 time points: (1) initial download/registration (approximately 200–300 megabytes), (2) recording of sessions (2–3 kilobytes/session), (3) push notifications (2–3 kilobytes/notification), and (4) periodic updates (20–30 kilobytes/update). Thus, our mHealth app platform incurs a very low data demand, which minimizes the barrier to use for participants.

#### Rationale for use of a control mHealth app

**Fig. 5:**
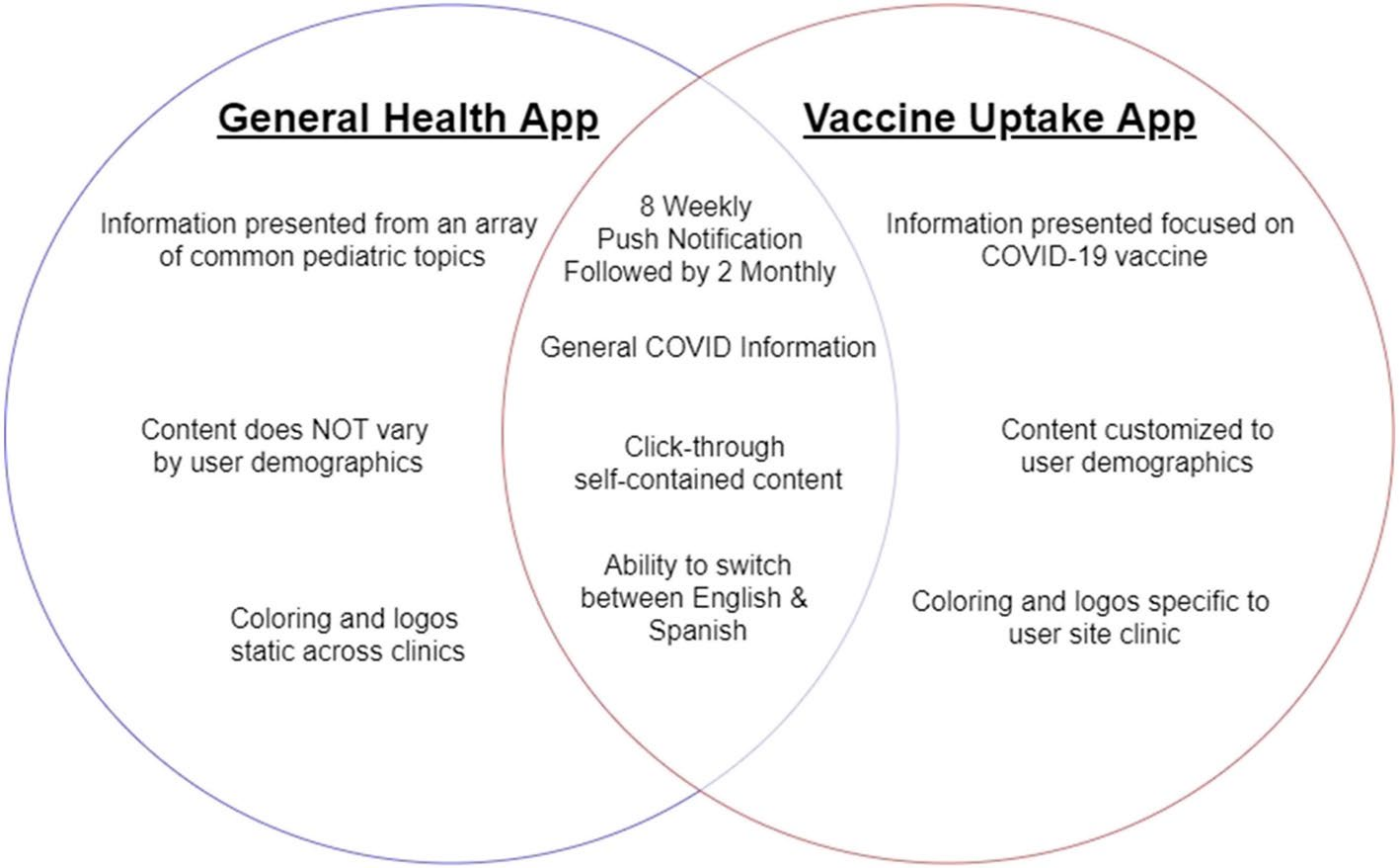
Comparison of functional and content elements of the Vaccine Uptake app and General Health app

Components of the General Health App and Vaccine Uptake App are summarized in Fig. 5. Participants in the General Health app arm will receive access to an mHealth app to ensure similarity in access to intervention materials (e.g., sending out of a link to register for app access via the same platforms) and barriers to access (e.g., low technology literacy, lack of an internet-capable device). The goal is to compare the impact of the customized, targeted content to generic information on an app, not the ability to access content on an educational app.

#### Limitations of using a mHealth control

App content, even if not customized or vaccine-related, may still elevate vaccination rates above what they would be with no intervention due to reducing barriers to accessing general information about infection control and prevention.

#### End of study definition

The study will end for the participant after they have reached 27 weeks of enrollment and either completed their 24-week assessment or failed to complete it by week 27.

### Study population

#### Inclusion criteria for participants

For this study, only parents/caregivers are participants. Parent/caregiver inclusion criteria are as follows:

##### Parent/caregiver inclusion criteria

1. Age of majority, as defined by the state of residency
2. Access to a mobile device that can store and run the study app for 24 weeks. Devices that can run the app include mobile phones and tablets running Android or iPhone Operating System (iOS) operating systems
3. Able to speak and read in English or Spanish
4. Be a parent/caregiver with primary medical decision-making and legal authority to consent to vaccination decisions for at least one child who meets the child inclusion criteria

Additionally, the parent/caregiver must have at least one child who:

1. Is age 6 months to less than age of majority, as defined by the child’s state of residence
2. Has not received any doses of COVID-19 vaccine based on parent/caregiver report
3. Is eligible to receive COVID-19 vaccine
4. Is a patient at a participating clinic

#### Rationale for parent/caregiver inclusion criteria

Parent/caregiver vaccine confidence/acceptance will vary widely, ranging from those who firmly refuse the COVID-19 vaccine to those who strongly endorse the COVID-19 vaccine for their children. All parents/caregivers, regardless of baseline vaccine hesitancy/confidence, are eligible to participate in the study. Subject randomization is intended to balance the proportion of vaccine refusing/vaccine endorsing participants. Parental vaccine hesitancy will not be assessed prior to enrollment. Such assessment may bias enrollment toward those individuals with greater vaccine acceptance through social desirability bias. Unlike vaccine-hesitant individuals, baseline vaccine refusing parents/caregivers are unlikely to be willing to participate in vaccine communication efforts and, therefore, will likely decline study participation during recruitment or informed consent [41].

Due to evolving COVID-19 EUAs by age categories and variable availability of vaccine by state, the study team may rescreen parents who do not meet enrollment criteria at initial screening.

### Inclusion criteria for clinic sites

Participating clinics will consent to use of local context (i.e., clinic logos, colors, local contact information, and pictures of clinic healthcare providers who consent to using their likeness) to create customized versions of the Vaccine Uptake app. All parents/caregivers enrolled in the study will be recruited based on their children receiving primary care at a participating clinic.

Participating clinics must meet all the inclusion criteria:

1. Provide primary pediatric general healthcare to at least 100 unique pediatric patients in the last 12 months; this can include family medicine clinics. Given that primary pediatric care in rural settings often occurs in family medicine clinics, including these clinics as potential performance sites will increase the generalizability of the findings
2. Have electronic health record (EHR) or billing database infrastructure to identify potential participants by reviewing the medical records of children within the study inclusion criterion of child-age range
3. Have ability to access their state’s COVID-19 vaccination registry
4. Meet at least one of the following diversity, equity, and inclusion criteria for the patient population served:
5.  < 60% non-Hispanic White;
6.  > 40% from a rural zip code as defined by rural–urban community area (RUCA) code ≥ 4 [43]; or
7.  > 40% with Medicaid/Medicare insurance or uninsured.

Sites are encouraged, but not required, to have a high Spanish-speaking population.

Sites are not required to stock and administer pediatric COVID-19 vaccines to serve as enrolling sites.

### Exclusion criteria

#### Parent/caregivers exclusion criteria

1. Has only a child or children with known contraindication to all COVID-19 vaccines
2. Has only a child or children whose other parent/caregiver is already a current or past participant in the study
3. Has a child or children enrolled in any other COVID-19 vaccine study of any kind
4. Past or present participation in a COVID-19 vaccine or behavioral trial
5. Has cognitive impairment that limits their ability to engage with the app content and/or make medical decisions regarding vaccination, based on the site investigator’s assessment and local human subjects research policies

#### Child exclusion criteria

1. Not a patient of a participating clinic
2. Prior receipt of least one dose of COVID-19 vaccine
3. Receiving or scheduled to receive COVID-19 vaccination at the time of parent/caregiver consent
4. Known medical contraindication to all COVID-19 vaccines
5. Ineligible to receive COVID-19 vaccine
6. Prior or current participation in a COVID-19 vaccine study of any kind

##### Rationale for parent/caregiver exclusion criteria

One or multiple parents/caregivers may have decision-making authority for a child’s vaccinations. Our primary outcome is the proportion of children who complete the COVID-19 vaccination primary series, not individual parental decision-making. Additionally, although only one parent/caregiver is enrolled, it is likely that information to which they are exposed during the course of the trial will be shared with another parent/caregiver who has decision-making authority if present.

### Lifestyle considerations

Participants will require sufficient access and literacy in technology to operate a mobile device and to download and interact with apps typically used on such devices. If participants have barriers in accessing sufficient Wi-Fi or mobile data bandwidth for initial app download (the only portion of app use anticipated to incur use levels above 1 mb), the enrollment visit will be arranged to take place somewhere with Wi-Fi to which the participant can freely connect.

If participants change or lose their phone or change operating systems, participants can contact the study team for technical support.

### Risk/benefit assessment

#### Known potential risks

This study poses minimal risk to participants (see definition of participants in Abbreviations & Definitions). Participants (in both the Vaccine Uptake app and General Health app arms) will provide protected health information (PHI) (e.g., children’s names, dates of birth, and dates of vaccination) to the study team. While the study team will make every effort to store this electronic information in secure electronic databases and physical documents in secure facilities, there remains a risk of accidental as well as mandated disclosure of PHI.

Participants will view content and answer questions related to the COVID-19 pandemic that may provoke some emotional stress or anxiety relating to the pandemic and/or COVID-19 vaccination.

The study will expose participants to content providing guidance in accordance with the most current recommendations from the CDC/ACIP. This information is subject to change. While educational content for both arms will undergo revisions in real time, participants may not always choose to access updated information after the study team posts it. In addition, app content will likely recommend vaccination with the COVID-19 vaccine while it is still available under FDA EUA rather than FDA approval for some of the participants based on their age.

#### Known potential benefits

Participants who receive the Vaccine Uptake app will receive self-directed, multi-format education on the COVID-19 vaccine, which may reduce participant stress and/or anxiety during decision-making regarding whether their child should receive the COVID-19 vaccine and increase their knowledge about vaccination. Caregiver participants who receive the Vaccine Uptake app will receive information on local resources for vaccinating their children against COVID-19, which may result in increased vaccination uptake among participants’ children. Caregiver participants who receive the General Health app will receive general health and infection prevention education. This may improve participants’ knowledge and risk-reduction behaviors, as well as lower their own and their children’s risk of contracting an infection and suffering the negative consequences from the infection. There is also potential for benefit to other parents and their children in the future if the study demonstrates that the intervention provides an effective means of educating parents about and increasing pediatric uptake of COVID-19 vaccine.

#### Assessment of potential risks and benefits

We do not anticipate significant health risks to participants and will make every effort to minimize the possible risks. The benefit of understanding the effect of a vaccine-education mHealth tool on promoting pediatric COVID-19 vaccination uptake outweighs the risks. Standards of medical care provided to participants’ children will not be altered based on participants’ study-related activities.

This protocol minimizes risks to participants by (a) correctly and promptly informing participants about risks so that they can join in partnership with the researcher in recognizing and reporting harms; (b) having staff properly trained in administering study procedures that may cause psychological distress, such as survey administration; and (c) providing safety monitoring.

If the FDA issues new warnings or precautions or withdraws EUA/approval for any of the COVID-19 vaccines for any of the included pediatric age groups during the study, the study team will take responsive action. Actions will include prompt notification of participants via at least one of the following methods: app push notification, text message, phone call, email, registered mail, and possibly study termination depending on the severity of the concern, as determined by the PIs and data safety monitoring board (DSMB).

### Recruitment, retention, consent, screening, and assessments

#### Recruitment

The target population will include all parents/caregivers meeting the inclusion criteria. Recruitment methods will prioritize enrolling participants from rural and underrepresented racial and ethnic communities, as these communities have experienced disproportionately lower vaccination rates and greater hardship due to the COVID-19 pandemic. To address the issue of potentially underperforming enrolling sites, we will ask ISPCTN sites to identify at least two clinics that can participate in the trial to achieve a goal of 24 participating clinics. In addition, enrolling sites will over-recruit by 15% to account for participant withdrawals. We will also initially allow competitive enrollment across clinics to enable high-performing enrolling sites to contribute maximally to achieving goal overall study enrollment.

Based on sample size estimates and weekly minimum recruitment goals, the goal accrual is 60 parent/caregiver participants per site across 15 enrolling sites with approximately 2 participating clinics per site. The anticipated accrual rate is a minimum of 7–8 participants per week per enrolling site over an 8-week recruitment period.

Recruitment for all enrolling sites will be competitive; there is no initial enrollment cap per enrolling site. Because of the compressed nature of recruitment, potential poor performance of enrolling sites must be addressed early in the recruitment process.

Site investigators and coordinators will meet weekly to review accrual. Sites will also receive weekly reports of enrollment performance, and high-performing sites will provide insights and support/mentorship for lower-performing sites with the help of Data Coordinating and Operations Center (DCOC) and protocol leadership. Sites will be prompted to share best practices as well as issues with recruitment during the weekly meetings. As part of recruitment, we will record how many subjects were approached, how many declined, and the reason for declining (e.g., out of age range, non-rural zip code, or declined) using a log developed by the enrolling site research staff. These logs will be monitored overall and by site.

At the end of each week of recruitment, the study team will assess accrual and make an explicit decision about adjusting recruitment strategies. Additional strategies to achieve goal recruitment will include:

1. Exten the recruitment window for all sites beyond the initial 8-week period (up to 6 months).
2. Targeted supplemental advertising through social media and local media outlets

##### Stopping rule

The project goal is to recruit at least 892 parents/caregivers over the up to 6-month recruitment period. At 3 months after study start, 20% of participants must be recruited. If recruitment does not meet this milestone, then the study PIs will report enrollment progress to the DSMB and obtain recommendations.

##### Initial recruitment methods

For efficiency, identification of potential participants will rely on several methods to capitalize on existing electronic health record databases and clinical workflows at study recruitment sites. Proposed identification methods include retrospective lists of recently seen patients and traditional advertisements for self-referral.

##### Retrospective recruitment

Site coordinators (or other designated personnel) can identify potential participants by reviewing a list of consecutively seen patients provided by participating clinics. The practice will develop the list from 12 months of patient-visit data (e.g., billing records). The site coordinator/designee will organize this list from more to less recent clinic visit dates and remove duplicate patients. The generated list will include patient zip code, race/ethnicity, and gender. Sites will be instructed to contact non-White and/or rural participants first to help ensure diverse participant enrollment. DCOC will provide a list of rural (RUCA ≥ 4) zip codes based on the 7/13/2019 revision of the 2010 RUCA Codes crosswalk [44].

##### Traditional recruitment

Sites may engage in traditional clinical trial recruitment methods focused on participant self-identification. Sites can recruit participants through various methods, including, but not limited to, provider recommendation at appointment visits, electronic health record messaging systems, and quick response (QR) codes. DCOC will provide a menu of options from which sites can choose, such as flyers, email, or advertisements on social media in English and Spanish. The DCOC will obtain institutional review board (IRB) approval for all recruitment materials. Recruitment materials will provide information to contact the site coordinator/designee for more information. During the consent process, site coordinators will ask participants recruited through the traditional method how they learned of the study.

Based on ECHO ISPCTN clinics’ recent experience with patient recruitment, we expect that the retrospective list method will have the highest yield. To allow enrolling site research staff to contact potential participants, we will send an opt-out/opt-in letter to potential participants, which will have the participating clinics’ logo and letterhead, and if possible, be signed by the lead clinicians. This letter will describe the study and inform families that research staff collaborating with the participating clinic will contact them. If families do/do not wish for the research team to contact them, the letter will ask participants to contact the enrolling site to opt-in for or opt-out of being contacted about the study. The letter sent to potential participants will include the following documents:

1. Opt-out/opt-in letter
2. Informed consent form (ICF) with detailed description of the study and with Health Insurance Portability and Accountability Act (HIPAA) language embedded (ICF + HIPAA)

##### In-person recruitment

This type of recruitment is possible only for those sites for which the research team is part of the participating clinic. The enrolling site research staff must be employed by the institution under which the participating clinic is affiliated, before engaging in any research-related activities. The staff of the participating clinics cannot be involved in any research-related activities.

##### Recruitment standardization

To ensure participant safety and consistent and high-quality data submission in compliance with protocol and study operations, the DCOC and study team will train enrolling site study staff. The DCOC expects all enrolling site study staff to take responsibility for learning the study, primarily by studying the protocol and manual of procedures (MOP), to ensure compliance with the protocol and good clinical practice (GCP) guidelines. The enrolling site study staff will receive training through the following methods: a mandatory training session in the form of an Investigator Meeting, and as needed training in the form of one-on-one enrolling site-specific training and/or other targeted training. DCOC personnel will train all enrolling site study staff on the protocol, reporting procedures for study deviations, potential study deviations, and noncompliance, as well as essential documentation for study conduct. The DCOC will train enrolling site study staff on in-person and tele informed consent processes and documentation.

Enrolling site study staff must document all training and provide the DCOC with this training documentation. The DCOC will train enrolling site study staff on any relevant study electronic data capture (EDC) system(s) to ensure that staff understand how to utilize the system to perform study-specific data collection and other necessary processes.

### Retention

#### User acceptance pilot testing

Prior to the start of participant enrollment, the apps for the Vaccine Uptake and General Health study arms will undergo pilot testing to optimize user acceptance. There will be approximately 5 testers from each of the following groups (total: 15):

1. Self-identified rural (English-speaking)
2. Self-identified non-rural (English-speaking)
3. Spanish-speaking

We will ensure that approximately 5 testers among the two English-speaking groups will not be non-Hispanic White. Each testing session will last 1 h and will involve reviewing user workflow and formative feedback on app content, including appropriateness of customizations by race, ethnicity, and rurality. User acceptance testing participants will receive $20 per test in which they participate.

#### Participant remuneration

Participants in the randomized trial will receive $20.00 for each assessment that is completed. There are four assessments; these will occur at baseline and weeks 8, 16, and 24. The total compensation that participants may receive is $80.00.

Study team activities to maximize participant retention include the following:

- Enrolling site coordinators will instruct participants to register in the app and will be available to refer participants to the Nebraska App Team if assistance is needed
- Research Team members will contact participants to confirm completion of app registration
- Scheduled push notifications during weeks 1–8 and then monthly for 2 months will prompt participants to engage with in-app content
- Ad hoc push notifications regarding national and local updates to vaccine availability/eligibility will prompt participants to engage with in-app content
- Enrolling site coordinators will contact participants at weeks 8, 16, and 24 for follow-up assessments
- Enrolling site coordinators will perform follow-up contact attempts for participants who do not complete their assessments at week 8, 16, or 24
- Contact methods for communicating with study participants will comprise multiple modalities, including (but not limited to) email, phone call, and/or text messages

### Screen failures and rescreens

#### Screening procedures and screen failures

For potential participants recruited with the retrospective method, site coordinators will contact (by phone or other methods) potential participants to briefly introduce the study. If potential participants are interested in learning more about the study, site coordinators will review the inclusion and exclusion criteria with potential participants to ensure they qualify. These data will be recorded by the research staff in the EDC. If potential participants are eligible, the site coordinator will perform the consent process. Potential participants should have received the ICF + HIPAA, and they should have had time to read the documents.

For potential participants recruited with the traditional method, site coordinators will ask participants if they would like to have a copy of the ICF + HIPAA form sent to them before discussing the study. If potential participants agree to discuss the study without a hard or electronic copy of the ICF + HIPAA, site coordinators will review the inclusion and exclusion criteria with potential participants to ensure they qualify. If potential participants are eligible, site coordinators will perform the consent process.

The study team will consider participants who undergo screening but do not enroll or consent to participate in the trial as screen failures, such as participants contacted by the research team but who do not meet inclusion and/or exclusion criteria.

Site coordinators must document and retain the following information for screen failures: basic demography, screen failure details, inclusion and/or exclusion criteria the participant did not meet, and other details as required. The data collected will be recorded by the research team in EDC. This data collection will be done before obtaining consent from the potential participants.

### Rescreenings

Site coordinators may rescreen parents/caregivers who initially do not meet enrollment criteria that are likely to change over time. Examples include parents/caregivers of children who age into the CDC/ACIP-recommended COVID-19 vaccination minimum age limit or who gain vaccination access due to progressing state prioritization schemes.

### Informed consent

This research study does not involve more than minimal risk of harm to participants and does not involve procedures for which written consent is normally required outside of the research context. The research study has been designed to perform all the study-related procedures remotely, and study personnel will not interact in person with potential participants during the intervention. The potential participants will be provided with a written statement regarding the research (ICF + HIPAA) before the consent process. The University of Arkansas for Medical Sciences (UAMS) IRB serves as the central IRB (cIRB), and they granted a waiver of consent documentation for adult participants (parents/caregivers).

Moreover, a full waiver of assent has been requested/granted. Children will not have to sign an assent and/or verbally agree to be part of this study. The ethical justification for requesting a waiver of assent is that this research study does not involve more than minimal risk of harm to participants and does not involve procedures for which written consent is normally required outside of the research context. Children participating in the study involve only medical chart review or access to the state registry to collect COVID-19 vaccination record.

#### Informed consent process

After determining that participants meet all inclusion criteria and none of the exclusion criteria, site coordinators or research team members will conduct the informed consent process to obtain a verbal consent. The study team anticipates that enrolling site coordinators/research team members will remotely conduct most consent processes (e.g., via telephone, or video conference platform). However, some in-person consenting could occur. The in-person consent will be done only by enrolling sites research staff members. Eligible potential participants should have received a copy of the ICF + HIPAA before being contacted by enrolling site coordinators or research team members. This will allow potential participants to follow the information presented by the site coordinators/research team members on their copy of the documents.

Informed consent is a process that starts before a participant agrees to participate in the trial and continues throughout the individual’s trial participation. Site coordinators/research team members will inform participants that participation is voluntary, that they may withdraw from the trial at any time without prejudice, and that nonparticipation will not adversely affect their medical care.

Site coordinators/research team members will explain the study to participants in terms participants can understand and answer any questions that may arise. The explanation will state the purposes, procedures, and potential risks of the study and describe participants’ rights as research participants. Participants will have the opportunity to carefully review the ICF + HIPAA and ask questions before consenting. Site coordinators/research team members will give participants the opportunity to discuss the study with their family or surrogates or think about participating in the study before agreeing to participate.

The enrolling site coordinators/research team member that performed the consent process must sign and date the ICF + HIPAA. Moreover, the research team member will have to document the informed consent process, following the current UAMS IRB policy. The consent process will be documented in a “Process Note.”

#### In-person consent

Enrolling site coordinators/research team members will coordinate with participating clinic staff to schedule a series of days to complete screening and consenting processes with previously contacted potential participants. If uncontacted, potentially qualifying participants visit the clinic during these days, site coordinators/research team members may screen and consent these individuals in person at the time of the visit or schedule a later time to do so. Site coordinators/research team members will ask participants how they learned of the study.

If clinic staff identify potential participants during a regular clinic or telemedicine visit, clinic staff can give, fax, or email potential participants study information (e.g., study pamphlet) and can give, fax, or email the potential participants’ contact information to the site coordinators/research team members. The site coordinators/research team members may contact potential participants to describe the study and, if participants agree, complete the consent process, including verifying eligibility.

### Assessments

#### Enrollment

At the end of the consent process, the enrolling site research team will complete the enrollment survey with participants. This survey will ask for the contact information of the participant (name, phone number, physical address, and email address) and self-identified rurality. The survey will ask for the name, race/ethnicity, sex, name of children’s primary care clinic and physician, and birthday of the participant’s children. The site coordinator/research team will record this information in the EDC.

#### App registration and usage (adherence)

During enrollment, site coordinators/research team members will ask participants if they would like assistance downloading and registering with the app. If participants request assistance, they will be scheduled with a Research Team member for technical assistance. After enrollment, caregiver participants will be block-randomized within their participating clinic by the enrolling site to either the Vaccine Uptake or General Health app study arms. A Research Team member will then register the caregiver in the assigned app using their assigned study number as well as the caregiver participant’s self-identified race, ethnicity, gender identity, rurality, and clinic of recruitment collected at enrollment. The Research Team will email caregiver participants instructions to download their assigned app. Once downloaded, participants will enter their email address to retrieve their registration. The Research Team will follow up with participants who have not used the app within 1 week of registration/instructions successfully sent via telephone, email, or video conference to confirm the completion of app registration. Each time the app is opened, every selection made within the app will be tagged and recorded with both the caregiver participant’s study number and a time date stamp. This data will be tracked throughout the caregiver participant’s 24-week intervention to determine adherence.

#### Baseline and follow-up assessments (week 8, 16, 24)

##### Baseline assessment

After enrollment and app registration, caregiver participants will be sent a link to the baseline survey with questions regarding their vaccine hesitancy, intent to vaccinate, perceived risks and benefits of the vaccine, who they trust for vaccine information, and disease avoidance behaviors. The survey will also ask some household-level questions, including:

- Highest education achieved by their children’s mother,
- Insurance status,
- Household members,
- Vaccination status of household members,
- COVID-19 infection status of household members,
- Increased infection vulnerability status (e.g., chronic health condition or other circumstances increasing vulnerability) of household members, and
- Experience of household members with any one of a set of common challenges (e.g., lack of food, housing).

All these questions, aside from vaccine hesitancy, will come from the Rapid Acceleration of Diagnostics in Underserved Populations (RADX-Up) Common Data Elements [45]. To assess vaccine hesitancy, caregiver participants will answer questions derived from the World Health Organization Strategic Advisory Group of Experts on Immunization (WHO SAGE) Vaccine Hesitancy Scale that will be adapted to COVID-19 vaccines [46].

##### Week 8 assessment

At the week 8 time point, the caregiver participants will be sent a link to an assessment that asks for the following topics:

1. *Vaccination status of eligible children*: The surveys will ask caregiver participants for the dates, brands, and administrators for all COVID-19 vaccinations that any of their children have received since enrollment, if any. For each vaccinated child, the survey will ask participants to upload a photo of the vaccination card. If a participant is unable to upload the photo or the writing on a card is illegible, the enrolling site personnel will contact the participant to schedule a time for a video conference to view and verify the card information.
2. *Usability of the app*: Surveys will include a systems usability assessment measured by the System Usability Scale (SUS). The SUS is a simple, ten-item scale giving a global view of subjective assessments of usability [47]. SUS instructions will ask caregiver participants to record their immediate response to each item, rather than thinking about items for a long time. Responses range from Strongly Agree to Strongly Disagree. The SUS yields a single number representing a composite measure of the overall usability of the system. SUS scores have a range of 0 to 100, with 100 representing a perfect score.

##### Week 16 assessment

Caregiver participants will complete an assessment at or around week 16. Table 1 lists the content of this assessment.

**Table 1:** Details the content covered in each assessment

	**Assessments**					
**Information collected**	**Enrollment**	**App**	**Baseline**	**Week 8**	**Week 16**	**Week 24**
**Children**						
Demographics	X					
COVID-19 vaccination status				X	X	X
**Participant (parent/caregiver)**						
Contact information	X			X		
Consent	X					
Demographics		X				
Vaccine hesitancy			X		X	
Intent to vaccinate			X		X	
Perceived benefits of vaccination			X		X	
Perceived risks of vaccination			X		X	
Trusted organizations			X		X	
Disease avoidance behaviors			X		X	
Perceived app usability				X		
Adherence		X				
**Household/family**						
Maternal education			X			
Insurance			X			
Vulnerable household members			X			
COVID-19 disease status			X		X	
COVID-19 disease history			X		X	
Challenges			X		X	

##### Week 24 assessment

At week 24, participants will be sent a link to an assessment that asks for the vaccine status of eligible children.

##### Participant vaccine status follow-up

Study staff will assess COVID-19 vaccination status as noted above. For any child whose status is not vaccination card-verified at the 8-, 16-, or 24-week assessment, site coordinators/study team members will review the child’s vaccination records in their clinic’s electronic medical record system and/or the state immunization registry beginning at week 25 period to determine COVID-19 vaccination status.

### Study intervention

#### Software, functionality, and content of the intervention

##### Overview

Participants in the Vaccine Uptake and General Health app arms will receive access to different mobile apps designed to provide health information. Content of the Vaccine Uptake app will focus on vaccine uptake, while content of the General Health app will focus on infection prevention and general child health.

##### App software

**Fig. 6:**
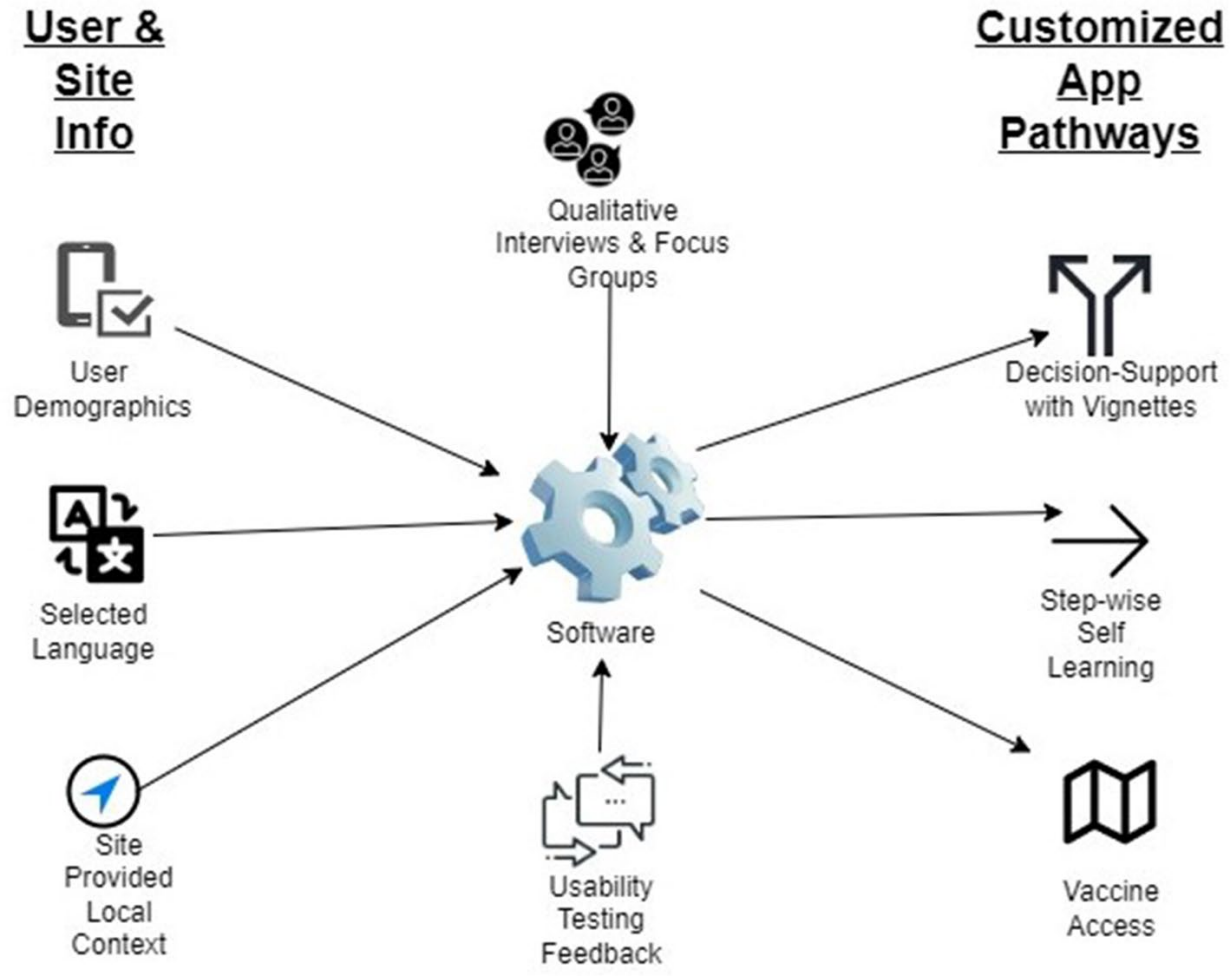
Schema of how individual user and site data contribute to the presentation of customized content in the Vaccine Uptake app

The Nebraska App Team will build the Vaccine Uptake and General Health apps using custom software created by Drs. Ellen Kerns and Russell McCulloh (protocol chairs) as a decision support tool. The software creates pathways (i.e., decision trees) by linking together a series of question cards in which the user’s response determines the next step. For the Vaccine Uptake App, the decision trees will be customized according to the user’s demographic information entered at registration, language preference (English or Spanish), and local context (state vaccination program stage/tier by age, local vaccination sites’ contact information, vaccination endorsement by local leaders and clinic staff). After the initial use session in which users complete their registration, the app will remember their registration characteristics and will render their tailored pathways accordingly for every subsequent use. Figure 6 outlines how individual user and site data contribute to the presentation of customized app content.

##### Control (general health app)

Specific elements of the General Health app will include:

1. *Push notifications*: The app will deliver messages directly to participants’ mobile devices prompting them to vaccinate their child and directing them to view specific app content (8 weeks with weekly push notifications; 2 monthly push notifications for 2 months [1 notification per month]).
2. *Content areas*: Specific topics presented as pathways are outlined in the schedule of push notifications (Table 2), but the general categories of topics included are:
  General infection prevention and mitigation measures
  General child health topics
  General COVID-19 information from the “How to Protect Yourself & Others” page for COVID-19 on the CDC website: https://www.cdc.gov/coronavirus/2019-ncov/prevent-getting-sick/prevention.html.
  Table 2 shows the schedule of push notifications during the study period

**Table 2:** Example vaccine uptake app components and HBM domains/vaccine hesitancy issues addressed

**Vaccine Uptake App Component**	**Example Component**^a^	**HBM domain**	**NIH CEAL Known Barrier/Concern**
Push Notifications, to include: -Behavioral nudges -Alerts to information about the vaccine in general -Clinic-specific information on vaccine issues	“Your child’s COVID-19 vaccine is available at [insert clinic name]. To schedule your appointment, touch here.” Links to local clinic’s appointment making resource “Did you know that over xx million children have received the COVID-19 vaccine? Hear from parents why they chose to vaccinate their children.” (Links to Vignette) “More than xx children have received the vaccine at your clinic. Click here to schedule your child’s vaccine appointment today.” “[Organization] is providing free rides to [insert vaccination site]. Click here for more information.” “COVID cases in children in your area are increasing. Protect your child with the vaccine today. Click here to schedule your appointment.”	Perceived barriers to vaccination Cues to action Psychological characteristics (peer group pressure) Perceived benefits Susceptibility to disease	Access issues/questions Do I need to pay for a COVID-19 vaccine?
Vaccine Access Pathway (“Find your child’s COVID-19 vaccine”)	Information about local events for vaccination; information about free rides to a vaccination site; links to schedule vaccine visits	Perceived barriers to vaccination Cues to action Access to care Psychological characteristics	Do I need an ID to get COVID-19 vaccine for my child? Do I (my child) have to be a legal resident to get COVID-19 vaccine? Do I need proof of insurance to get the COVID-19 vaccine for my child? Do I need to provide personal identifying information (address, phone number, etc.) to get my child vaccinated?
Vignettes/step-wise decision support. Will include: -Relatable storytelling -Testimonials from local staff/leaders	Vignette: Taking your teenager to get vaccinated Vignette: Why I vaccinated my child Vignette: Parent of child with COVID-19/MISC. Their story Vignette: Provider testimonial (trusted messenger) on long COVID/MISC and the choice to vaccinate their own children Vignette: School outbreak, from the perspective of teachers/students/parents Vignette: Student athlete who got COVID Vignette: Protecting a vulnerable family member	Health motivation Safety/perceived severity of effects from the vaccine Psychological characteristics Perceived benefits Perceived susceptibility Perceived susceptibility Perceived severity of SARS-CoV-2 Psychological characteristics Perceived susceptibility Perceived severity of SARS-CoV-2 Perceived benefits of the vaccine Health motivation Cues to action Perceived susceptibility Perceived severity of SARS-CoV-2 Perceived benefits of the vaccine Cues to action Perceived susceptibility Perceived severity of SARS-CoV-2 Perceived benefits of the vaccine Psychological characteristics Perceived benefits of the vaccine Health motivation Cues to action	Does the COVID-19 vaccine impact fertility? How effective is the COVID-19 vaccine? Does COVID-19 cause disease in children? Is it worse than the flu? Are COVID-19 vaccines safe? How were COVID-19 vaccines developed? Does COVID-19 infect children aren’t they too healthy to get sick? Is natural immunity just as good as getting vaccinated? Does receiving the vaccine give you COVID-19? If you are vaccinated, then why do I have to get vaccinated?
Customization for clinic site	Clinic logos/colors Clinic location/contact information Links to clinic’s website and appointment-making tools Clinic-specific vaccination events (when applicable)	Perceived barriers to vaccination Psychological characteristics Cues to action (trusted organization) Health motivation (trusted organization)	Questions around trust and trusted messengers
Customization for participant socio-demographics	Vignette pictures/content adjusted for race, ethnicity, language, and/or rurality	Psychological characteristics Susceptibility to disease Benefits of vaccination Health motivation	Does the vaccine work for people like my child? Has the vaccine been tested in people like my child?
Self-directed learning pathways	COVID-19 by the numbers (stats and pictographs) Natural immunity vs vaccination Frequently asked questions about COVID-19	Susceptibility/severity of infection Benefits of vaccination Benefits of vaccination Susceptibility/severity of infection Potentially multiple avenues, depending upon user motivation to access information	Various questions, concerns, and misinformation topics, with focus on topics from CEAL, CDC, and qualitative interviews

*HBM* Health belief model

^a^Specific content may vary based on user acceptance testing and qualitative study results

##### Intervention exposure (vaccine uptake app)

The intervention app will consist of 3 pathways: (1) step-wise decision support with narrative vignettes, (2) self-directed learning on each selected topic area, (3) geographic location-specific vaccine access information and corresponding push notifications. The Health Belief Model will drive topics for the vignettes and self-directed learning. Specific content is guided by the qualitative interviews previously conducted in an ongoing study and include (among others identified): Safety; Effectiveness and personal/family susceptibility/vulnerability; Benefits (personal, family, community); Trust, decision-making/autonomy; Availability/accessibility. The list below provides specifics of the pathways and push notifications:

- *Step-wise decision support with narrative vignettes*: Using this pathway, participants will view information about COVID-19 vaccination in the form of short stories that include images of children, healthcare providers, and family members, as appropriate. The study team will tailor images and text content of the narrative vignettes to participant registration demographics. For instance, rural White adult participants with teenage children will mostly view vignettes featuring rural, White parents, teens, and healthcare providers discussing issues noted in the qualitative study to be of interest to this demographic group. Similarly, Hispanic participants of infants will mostly view vignettes featuring Hispanic parents, infants, and healthcare providers discussing issues of interest to this demographic group, etc. As everyone benefits from gaining input from diverse sources, pathways will not exclusively feature messengers with complete demographic concordance. Participants can review other vignettes of interest to them or explore different questions within vignettes through in-app navigational buttons.
- *Step-wise self-directed learning*: Using this pathway, the app will give participants a menu of topics. Clicking on any of these will provide the participant general information on the topic and external links derived from the CDC and local health authorities, including hyperlinks that will open an internet browser window within the app. Unlike a website frequently asked question page, users will access information through branching logic based on topic area.
- *Vaccine access*: Using this pathway, the app will give participants local vaccine availability, recommendations for vaccination (e.g., at what age children can receive the vaccine locally), links to sign-up for a vaccination appointment locally, and resources for traveling to vaccination sites, etc.
- *Push notifications*: The app will deliver messages directly to participants’ mobile devices through 8 weekly and 2 monthly push notifications. These messages will direct participants to view specific app content (scheduled once weekly and once monthly, see Table 2 for topic schedule) and alert participants to local or national changes to vaccine availability or vaccination recommendations (as necessary, up to once weekly).
- *Content areas*: The study team will use the Health Belief Model (Fig. 1) to derive general content, and the qualitative study interviews will guide specific content, including (among others identified):
  - Vaccine safety
  - Vaccine effectiveness
  - Personal/family susceptibility/vulnerability to COVID-19 disease morbidity and mortality
  - Benefits of vaccination (personal, family, community)
  - Trust in government, healthcare systems regarding vaccination; decision-making/autonomy in choosing vaccination for children
  - Vaccination availability/accessibility

##### Communication addressing vaccine hesitancy in the vaccine uptake app

A previously completed qualitative study will use focus groups of specific sociodemographic groups in four states to understand the communication sources people rely on and misinformation influencing COVID-19 vaccine decision-making. The mHealth app will use principles outlined by NIH Community Engagement Alliance (CEAL) against COVID-19 as we construct the pathways (Table 2) [9, 48]. The focus groups will identify key misinformation, which will be used in each pathway. For example, in the vignettes there will be testimonials from their clinic staff, and the step-wise decision-making will present accepted facts without reinforcing misinformation [49, 50].

#### Measures to minimize bias: randomization

##### Randomization

The study will use a 1:1 parallel design at the caregiver level to assign caregivers to the intervention (Vaccine Uptake app) or the control (General Health app) arm. The study team will stratify randomization by participating clinic and in varying block of participants using a permuted design. Randomization will occur after enrollment and before the baseline assessment.

#### Study intervention compliance

The study team will measure and monitor compliance using app usage analytics. The study team will summarize app usage measures as follows:

- Cumulative number of times app was accessed (sessions) to determine total content exposure
- Average session duration
- Median weekly app sessions to determine rate of engagement over time
- Number of unique content areas are accessed within the app
- Whether participants allowed receipt of push notifications

To be compliant to the intervention, participants must have at least one app session in which they access content from at least one app pathway.

### Study intervention discontinuation and participant discontinuation/withdrawal

#### Participant discontinuation/withdrawal from the study

The study team will consider the following to be an early-terminated participant: any participant who completes the informed consent process and enrolls in the study but later withdraws their consent, or the site investigator removes the participant from the study. The study team will not replace early-terminated participants; instead, sites will over-recruit to account for early-terminated participants.

Participants may withdraw from the study at their own request, or the site investigator, DSMB, and/or DCOC can withdraw a participant. If a participant withdraws from the study, site coordinators or investigators can ask the participant why they chose to withdraw. Site coordinators will document the reasons for withdrawal.

#### Lost to follow-up

The study team will consider participants lost to follow-up if they do not have vaccination status confirmed by the end of the study period (either survey/self-report or via EMR/registry check).

To minimize the number of participants lost to follow-up, the study team will send survey links and notify participants beforehand, asking participants to respond to the survey link. Survey reminders will be sent to non-respondents daily for 3 days. Site coordinators will then contact any remaining non-respondents.

### Study assessments and procedures

#### Efficacy assessments

The *primary endpoint* is child initiation and completion of COVID-19 vaccination by the end of week 24 post caregiver randomization. Verification of vaccine receipt will occur by any of the following methods:

1. Photograph of the child’s vaccine card, furnished by the caregiver
2. Medical record documentation of child vaccine receipt
3. Recorded vaccine receipt in the appropriate state vaccine registry

*Secondary endpoint #1* is child receipt of ≥ 1 dose of the COVID-19 vaccination series. Verification of vaccine receipt will be the same as for the primary endpoint.

*Secondary endpoint #2* is enrolled caregiver domain scores on the modified SAGE Vaccine Hesitancy Scale adapted for the COVID-19 Vaccine from baseline and the end of week 16 post caregiver randomization. Caregiver participants will complete this survey electronically.

#### Safety and other assessments

Because this study is of an educational intervention and poses minimal risk, adverse events (AEs) and serious adverse events (SAEs) are not expected. The study team will not solicit AEs or SAEs but will provide all participants with a site-specific telephone number to report AEs/SAEs. The study team will track severe and serious AEs that are potentially study related.

##### Adverse events and serious adverse events

**Definition of adverse events (AE)**

An AE is defined as any untoward occurrence associated with the use of an intervention in humans, whether or not considered intervention related (21 CFR 312.32 [a]). The study team will not solicit AEs or SAEs but will provide all participants with a site-specific telephone number to report AEs/SAEs. The study team will document severe AEs and SAEs and will track SAEs.

**Definition of serious adverse events (SAE)**

We will consider an AE or suspected adverse reaction “serious” if, in the view of the PIs, medical monitor, DSMB, or sponsor, it results in any of the following outcomes:

- Death
- Life-threatening AE
- Inpatient hospitalization or prolongation of existing hospitalization
- Persistent or significant incapacitation or substantial disruption of the ability to conduct normal life functions
- Congenital anomaly/birth defect

Important medical events that may not result in death, be life-threatening, or require hospitalization may be considered serious when, based upon appropriate medical judgment, they may jeopardize the participant and may require medical or surgical intervention to prevent one of the outcomes listed in the SAE definition. Examples of such medical events include allergic bronchospasm requiring intensive treatment in an emergency room or at home, blood dyscrasias, or convulsions that do not result in inpatient hospitalization, or the development of drug dependency or drug abuse.

##### Classification of an adverse event

**Severity of event**

These are the guidelines to describe the severity of AEs:

1. *Mild*: Events require minimal or no treatment and do not interfere with the participant’s daily activities.
2. *Moderate*: Events result in a low level of inconvenience or concern with the therapeutic measures. Moderate events may cause some interference with daily functioning.
3. *Severe*: Events interrupt a participant’s usual daily activity and may require systemic drug therapy or other treatment. Severe events are usually potentially life threatening or incapacitating. Of note, the term “severe” does not necessarily equate to “serious.”

**Relationship to study intervention**

All severe AEs and SAEs must have their relationship to trial intervention assessed by the clinician who examines and evaluates the participant based on temporal relationship and her/his clinical judgment. We will grade the degree of certainty about causality by using the categories below.

1. *Related*: We know the AE occurred with the trial intervention, there is a reasonable possibility that the trial intervention caused the AE, or there is a temporal relationship between the trial intervention and event. Reasonable possibility means that there is evidence to suggest a causal relationship between the trial intervention and the AE.
2. *Not Related*: There is not a reasonable possibility that the administration of the trial intervention caused the event, there is no temporal relationship between the trial intervention and event onset, or there is an established alternate etiology.

##### Expectedness

The site investigators will be responsible for determining whether a severe AE or SAE is expected or unexpected. A severe AE or SAE will be considered unexpected if the nature, severity, or frequency of the event is not consistent with the risk information previously described for the trial intervention.

Potential events could include the following.

- Anxiety
- Depression
- Feeling stigmatized or threatened

##### Time period and frequency for event assessment and follow-up

The study team will provide participants with a site-specific telephone number to report severe AEs or SAEs. Since this study will not administer the COVID-19 vaccine or any other intervention beyond the app, the study team will not actively solicit AEs or SAEs or monitor any events related to COVID-19 vaccination. When site coordinators or site investigators learn of a severe AE or SAE, they will refer participants to their primary care physician and will record the severe AE or SAE into the EDC and will track the SAE.

Site coordinators/research team members will record severe AEs as described in the next section and in the (MOP). Severe AE data collection typically includes event description, time of onset, if available, resolution time and day, if available, and the site investigator’s assessment of severity, relationship to study participation or intervention (assessed only by those with the training and authority to make a diagnosis), and time of resolution/stabilization of the event. Site coordinators/research team members must appropriately document severe AEs occurring during the trial, regardless of relationship to the trial. The study team will track severe AEs until one of the following criteria are met: resolution, the condition stabilizes, the event is otherwise explained or is judged by the site investigator to be no longer clinically significant, or the participant withdraws from the study. The study team will track all SAEs by asking site coordinators/research team members about the SAE every 14 days until resolution or until the site investigator determines the event is chronic or until the participant is stable. The DCOC may request other supporting documentation of the event, and site investigators or site coordinators should provide this documentation as soon as possible.

Site coordinators/research team members will document changes in a severe AE in the source document and EDC to allow for an assessment of the duration of the event. Severe AEs characterized as intermittent require documentation of onset and duration of each episode.

Participants may report severe AEs for 7 days after completing all study procedures and SAEs for 30 days after completing all study procedures. Site coordinators and/or DCOC safety personnel will record and track according to this protocol and DCOC standard operating procedures (SOPs).

##### Adverse event reporting

Severe AEs will be included in the statistical analysis, and site coordinators/research team members will document severe AEs in the EDC system and follow the reporting procedures outlined in the MOP. The MOP section on documenting severe AEs will encompass the requirements of the:

1. cIRB policies and procedures
2. SOPs for the Streamlined, Multisite, Accelerated Resources for Trials IRB Reliance platform (SMART IRB)
3. ICH E6(R2), GCP: Integrated Addendum to ICH E6(R1): Guidance for Industry
4. Local IRB policies and procedures, when applicable. If there are discrepancies between the procedures, the most stringent of the procedures will be followed.

The DCOC will report severe AEs to the cIRB for its yearly continuing review. The DCOC will report to the central IRB (cIRB), as specified in the study-specific IRB communication plan, based on recommendations of the SMART IRB. Sites will also report severe AEs to the local IRB, per local IRB policies and procedures, and make them available to the sponsor (DCOC) on a continuing basis through the EDC system. The study team will report to the DSMB, per the DSMB charter.

The trial-specific MOP will describe the details of the reporting structure and additional details related to timelines for reporting.

The specific regulations of the FDA do not apply to this protocol because this is not an FDA-regulated trial.

##### Serious adverse event reporting

Site coordinators/research team members will document all SAEs in the EDC, and the DCOC will report SAEs, per Table 3 below. The DCOC will notify monitoring personnel (e.g., medical monitor) and monitoring bodies (e.g., DSMB), per the DSMB charter.

**Table 3:** SAE reporting

Report to	Timing	Notes
cIRB (SMART IRB definition)	Yearly (minimum)/at continuing review	For continuing review; Submitted to IRB via DCOC
Local IRB	Per local IRB policies and procedures	
DCOC (Sponsor, per SMART IRB definitions, and lead trial team)	Immediately, but no later than 48 h after finding out about the event	CO-SOP-012.v1.0/SOP
Overall PIs (per SMART IRB definitions)	Immediately, but no later than 24 h after finding out about the event	
Medical Monitor	Per DSMB charter	
DSMB	Per DSMB charter	

The study team will track all SAEs until satisfactorily resolved or the site investigator deems the event is chronic or the participant is stable. The DCOC/trial sponsor may request other supporting documentation, and sites should provide this as soon as possible.

The DCOC will ensure the summary is accurate and will report SAE data to the cIRB in time for consideration at the next continuing review. The DCOC will also create and provide any SAE data summaries requested by the monitors or specified in the DSMB charter. The site investigators will be responsible for following their local institution’s requirements.

##### Reporting events to participants

The study team will notify participants of those trial-related (or potentially trial-related) SAEs that may affect their willingness to continue with the trial or their future health. Any of the following can determine if study personnel should contact participants: the IRB, the medical monitor, the DSMB, or the PIs. The person or oversight body that makes the determination will inform the DCOC, which will instruct the site investigators and site coordinators to contact the participants consented through their site.

Site coordinators/research team members will record any contact with participants, if necessary, in the EDC and/or trial log.

#### Unanticipated problems

##### Definition of unanticipated problems (UP)

The Office for Human Research Protections (OHRP) considers unanticipated problems involving risks to subjects or others (UPIRTSOs) to be any problem, event, or new information that is:

- Unanticipated or unexpected;
- Related to the research; and
- Involves new or increased risks to subjects or others.

A UPIRTSO is not necessarily an AE or an SAE. For example, a breach of confidentiality is a potential UPIRTSO that is not an AE or SAE.

##### Unanticipated problem reporting

Site coordinators/research team members must report potential UPIRTSOs, per this protocol, to the cIRB, via the method(s) specified in the MOP, and directly to the DCOC. If the cIRB determines the issue is, indeed, a UPIRTSO, the DCOC will notify persons and entities as required by cIRB policies and procedures, the study-specific communication plan, and the DSMB charter. Site coordinators/research team members must also report these events to their local institutions according to the rules and regulations of the local institution. The cIRB will report the issue to OHRP, per the policies of the cIRB. The cIRB required reporting times are provided in the contemporaneous version of 10.2, *Information that must be reported to the IRB and IRB actions*, which is available via http://irb.uams.edu/irb-policies/current-irb-policies/principal-investigator-responsibilities/. The reporting times, at the time of the approval of this protocol, are in Table 4 below.

**Table 4:** Unanticipated problem reporting (per UAMS IRB policy at time of approval of this protocol)

Unanticipated problem	Required reporting time to cIRB
Death or life-threatening	Immediately to IRB office or IRB Chair
All other events	Within 10 days of event or notification of event if non-local

##### Reporting unanticipated problems to participants

For reporting UPIRTSOs to participants, we will follow the same procedures as outlined under “Reporting events to participants”.

### Statistical considerations

#### Statistical hypotheses

Our hypothesis for our primary endpoint is that unvaccinated children of caregivers assigned to the Vaccine Uptake app will be more likely to achieve COVID-19 vaccine series completion than those children whose caregivers are assigned to the General Health app. The null hypothesis is that the vaccination series completion rate among children of caregivers assigned to the Vaccine Uptake app will not differ from the vaccine series completion rate among children of caregivers assigned to the General Health app.

#### Sample size determination

The primary outcome for this study is the proportion of unvaccinated children who complete the COVID-19 vaccine series during the study period. The underlying assumption, based on available data for ISPCTN site states, is that without the mHealth intervention, the COVID-19 vaccine uptake would occur in 30% of children [16].

##### Justification of primary outcome effect size

Table 5 summarizes vaccination rates among children 12–17 years old through July 31, 2021, as published by the CDC [51]. Based on these data, we see that vaccination rates decline with decreasing age and that ECHO ISPCTN states demonstrate a trend toward lower overall and by-age vaccination rates. When excluding Vermont, Rhode Island, and New Hampshire (states that are part of the highest regional vaccine uptake rates in the USA), the other 15 ECHO ISPCTN states have vaccination rates significantly lower than non-IDeA states. Assuming that cumulative vaccination rates continue to increase over time and that vaccination rates for children < 12 years old will continue the trend toward lower vaccination rates, we anticipate that 30% overall vaccination rates among eligible children at the time of study launch is reasonable.

**Table 5:** Proportion of children 12–17 years old who completed COVID-19 vaccination by July 31, 2021

	12–17 Med[IQR]	16–17 years old	14–15 years old	12–13 years old
Overall	30% [25–42%]	40% [32–51%]	29% [24–41%]	24% [19–34%]
IDeA state residents	24% [20–37%]	33% [26–49%]	23% [19–35%]	20% [14–32%]
IDeA state residents (excluding New England)	23% [19–36%]^a^	31% [26–45%]	22% [18–29%]	19% [14–31%]
Non-IDeA state residents	32% [25–42%]	41% [32–51%]	29% [24–41%]	25% [19–34%]

^a^Difference from non-IDeA state residents (*p* = 0.028)

We based the 10% increase in vaccination rates on the cumulative estimated effects of intervention components within the Vaccine Uptake app. As discussed in the “ Background”, behavioral nudges such as text messages (i.e., push notifications within the app) are associated with a 5% increase in vaccination rates [52]. App-based education and communication has shown variable increases in vaccination rates and intent to vaccinate, up to 8–10% [53, 54]. Thus, a combined 10% increase in vaccination rates is reasonable to expect from the Vaccine Uptake app since it combines multiple approaches in the same intervention.

In determining sample size, each caregiver is considered to be a cluster with an average of 2 unvaccinated children. To demonstrate an improvement in the proportion of unvaccinated children of caregivers who complete the COVID-19 vaccination series due to the mHealth intervention from 30 to 40% [27, 55, 56] would require 758 caregivers (379 per arm), at the two-sided 0.05 significance level with power of 0.90 and an intraclass correlation coefficient of 0.60. Table 6 below shows the sample size needed to have 90% power to detect a 10-percentage point increase in the proportion of children who complete the vaccination series using the Vaccine Uptake app with varying levels of intraclass correlation. To ensure that there are at least 758 caregivers, the primary analysis population, we expect to randomize 892 caregivers to account for up to 15% of randomized participants who do not use the Vaccine Uptake app or General Health app after downloading it during enrollment.

**Table 6:** Sample size calculations based on ICC

Intraclass correlation coefficient	Sample size
0.40	331 per arm (662 total)
0.50	355 per arm (710 total)
0.60	379 per arm (758 total)
0.70	402 per arm (804 total)
0.80	426 per arm (852 total)

#### Populations for analyses

This study will have three analysis populations:

1. ITT population—This population will include all participants randomized into the study.
2. Modified ITT (mITT) population—This population will include all participants who are randomized into the study, were eligible for the study, and completed at least one session in the app involving the use of at least one pathway.
3. Per-protocol (PP) population—This population will include all participants in the mITT population who were maintained in the study for the full 27 weeks.

The primary population for analysis will be the ITT population. Study statisticians will perform additional analyses on the PP and mITT populations.

#### Statistical analyses

##### General approach

Following finalization of the protocol, but prior to data lock, the DCOC statistical team will issue a Statistical Analysis Plan (SAP) as a separate document, which will provide detailed analytical plans for the set of analyses outlined below. The statistical team will conduct all statistical analyses following the statistical principles for clinical trials as specified in ICH Statistical Principles for Clinical Trials (ICH Topic E9). The study team will describe and justify any deviations from the planned analyses in the final integrated clinical study report. The study team will present all study data and summary tables for the overall study and by study sites.

The statistical team will summarize descriptive statistics for continuous data by using mean and standard deviation or median and interquartile range, as appropriate. The team will also summarize categorical data by using frequency and percent, and they will investigate any outliers detected during data review and will define in the SAP methods for handling outliers or data transformation.

##### Analysis of the primary efficacy endpoint

**COVID-19 vaccine series completion**

For each vaccine-eligible child, the primary endpoint will be whether or not the child initiates and completes the COVID-19 vaccine series during the 24 weeks of the intervention period. The study team will define vaccine series completion as per the current ACIP guidance for the vaccine product. For children who receive a product that requires more than 2 doses for the primary series, receipt of up to 3 doses will be considered complete. Vaccine doses will only be valid for study purposes if given within the 24 weeks of study participation. Additional doses, when required for primary series completion, will only be valid if they are in accordance with Advisory Committee on Immunization Practices (ACIP)-recommended interval minus a 4-day grace period, in accordance with recommendations of the ACIP. Also, per ACIP, there will be no maximum interval between valid doses. Incorrect second vaccine product (i.e., mixed series) will be invalid for study purposes. The statistical team will use a mixed model with the binomial distribution and the logit link to compare the two intervention groups with respect to the proportions of children who complete COVID-19 vaccination, using a site random effect and controlling for clustering by caregiver.

##### Analysis of the secondary endpoints

**Vaccine series initiation**

For each vaccine-eligible child, this secondary endpoint will be defined as whether or not the child initiates the COVID-19 vaccine series during the 16 weeks of study participation. Vaccine series initiation will be receipt of at least 1 valid dose of any COVID-19 vaccine product. Vaccine doses for series initiation will only be valid for study purposes if given within the 16 weeks of study participation. The statistical team will use a mixed model with the binomial distribution and the logit link to compare the two intervention groups with respect to the proportions of children who initiate COVID-19 vaccination, using site as a random effect and controlling for clustering by caregiver.

**Parental attitude toward pediatric COVID-19 vaccination**

The study team will evaluate parental attitude toward pediatric COVID-19 vaccination by using the vaccine hesitancy questionnaire that includes ten statements with ordinal responses using a 5-point Likert scale. At baseline and week 16, the study statisticians will generate summary statistics for each of the 10 questions for the two intervention groups. Similarly, the change in responses from baseline to end of study will be determined for each intervention group. The study statisticians will use the general linear mixed model to evaluate the intervention effect on responses to each measure at baseline and end of study, and they will use this model to evaluate the change in each measure by using site as a random effect. Within each intervention arm and for each statement, the study statisticians will use the Wilcoxon signed rank test to determine if the change in response from baseline to end of study is significantly different from zero.

##### Safety analyses

The DCOC will detail reported intervention-related severe AEs and SAEs experienced by participants and will summarize these by the study arm. The DCOC will present summary statistics for overall and by sites.

##### Planned interim analyses

There are no planned interim analyses for this study.

##### Sub-group analyses

If there are enough study participants in specific racial/ethnic categories and or urban/rural and/or age group categories, the study team will perform the planned analyses for the primary efficacy analyses within subgroups.

##### Sensitivity analyses

While some participants may choose not to use the app beyond the initial download session, their outcomes will still be assessed and used to determine efficacy of the intervention. App interactions—or lack thereof—will be informative to understanding how participants engage with delivered content and how those interactions—or lack thereof—correlate with primary and secondary endpoints. The statistical team will use logistic regression analyses to determine the association between mHealth usage levels and completion or initiation of COVID-19 vaccine series.

##### Tabulation of individual participant data

The statistical team will provide a detailed description of participant disposition, will tabulate the number of enrolled participants overall and by study site, and will present this data as counts and percentages. Additionally, the statistical team will summarize the number of participants either completing or discontinuing the study using counts and percentages.

##### Exploratory analyses

The statistical team will use logistic regression analyses to determine the association between demographic factors including rurality and completion or initiation of COVID-19 vaccine series.

### Supporting documentation and operational considerations

#### Regulatory, ethical, and study oversight considerations

##### Study discontinuation and closure

The trial may be suspended or stopped per any stopping/suspension specifications in the DSMB charter. The cIRB may also stop or suspend the trial. Early termination of the study may be permanent if there is sufficient cause.

The suspending or terminating party will provide, directly or indirectly, written notification documenting the reason for trial suspension or termination to the following, as applicable: trial participants, PIs, site investigators, cIRB, local IRBs, NIH, DCOC, and OHRP. Persons and offices notified will include those specified in the trial MOP, the cIRB’s policies and procedures, and the SMART IRB policies and procedures.

The suspending or terminating party will also contact trial participants and inform them of any changes to the trial visit schedule. Circumstances that may warrant trial termination or suspension include, but are not limited to:

- Determination of unexpected, significant, or unacceptable risk to participants
- Insufficient compliance to protocol requirements
- Data that are not sufficiently complete and/or evaluable

If the trial is temporarily suspended, it may resume once concerns about safety, protocol compliance, and data quality are addressed and are satisfactory to the DSMB, the cIRB, the local IRB(s) (when applicable), the funding agency, and the DCOC.

##### Confidentiality and privacy

The study team will record data from the software to a HIPAA-compliant cloud PostGres Azure database. Sites and study team members will conduct all trial activities in as private a manner as possible.

Records will be maintained as required by the privacy and security rules promulgated by the HIPAA (Title 45 of the CFR Part 164) [21, 57].

During the trial, site investigators and/or site coordinators will keep all trial records in secure locations that only authorized personnel can access. Examples of secure locations include but are not limited to (1) locked file cabinet(s) in a limited (badge or key) access room, or (2) password-protected computer systems. Study personnel may only transmit records that contain PHI, as defined by HIPAA, through an open email system if the personnel encrypt the data. Password protection alone is insufficient for data transmission through an open email system (e.g., Outlook). After trial completion, access to trial records will be limited (see next section).

Certain bodies/institutions may need to review information, including the participant information, for any of the following reasons: to process information or to ensure compliance with the protocol and other applicable requirements (such as the policies and procedures of the cIRB). Institutions/bodies that may have access to the participants’ information include:

- UAMS IRB (cIRB) and other oversite offices
- IRB for the site through which the participant is consented
- OHRP
- DCOC
- NIH

Individuals with access to trial records will be:

- Study PIs
- Site investigators
- Site coordinators
- Data managers at enrolling site(s)

##### Study governance

The clinical trial outlined in this protocol is part of the ECHO ISPCTN, a branch of the program supported by the NIH.

The data coordination, technical instruction, data standards, quality control (QC) and quality assurance (QA), and operational coordination for the clinical trial protocol outlined here (and for the ECHO ISPCTN overall) is provided by the DCOC.

The Steering Committee governs the ECHO ISPCTN and includes representatives from all site awardees, as well as representatives from the DCOC and the NIH. Overseeing the work of the Steering Committee is the NIH ECHO office, as well as an executive Leadership Committee.

A team of content experts and DCOC staff completed this protocol, and the ECHO ISPCTN Steering Committee reviewed it. The NIH Protocol Review Committee and the DSMB further reviewed the protocol.

##### Safety oversight

Ensuring participant safety is the responsibility of all study team members, especially the PIs, site investigators, site coordinators, and monitors. A medical monitor and the DSMB will provide oversight.

The medical monitor will be a pediatrician with expertise in infectious disease and vaccinations and will be independent of the trial. The NIH will convene the DSMB, and it will meet regularly, according to its charter.

The entities that will receive reports include, but are not limited to, the DCOC and the NIH. The DSMB charter will provide additional information.

##### Clinical monitoring

We will conduct enrolling site monitoring to ensure that site investigators and site coordinators are protecting the rights and well-being of trial participants, that the reported trial data are accurate, complete, and verifiable, and that the conduct of the trial complies with the currently approved protocol/amendment(s), with ICH GCP, and with applicable regulatory requirement(s).

- A DCOC team member or designee will remotely monitor enrolling sites to ensure data quality and integrity via weekly phone meetings and queries of EDC entries.
- On-site monitoring visits will also be performed at each site, per the Site Monitoring Plan, if needed for cause.
- The DCOC will conduct an enrolling site close-out visit at the end of the study.
- Clinical site monitors will document details of their activities and findings, per the Site Monitoring Plan. The Site Monitoring Plan describes the monitoring details (i.e., who will conduct, at what frequency, at what level of detail, and distribution of monitoring reports).

##### Quality assurance and quality control

Each IRB-approved site entering data will perform internal quality management of study conduct, data collection, documentation, and completion. Each site will follow the MOP and any additional written site-specific SOPs. The operating procedures will include, but are not limited to, procedures for (1) performing the consent process; (2) data collection, entry, review, and submission processes; (3) assigning roles and responsibilities of site personnel; and (4) training methods for study staff. Sites will provide direct access to all their facilities, source data/documents, and reports for the purpose of monitoring and auditing by the DCOC and inspection by local and regulatory authorities. When electronic health records are source data/documents, sites must provide read-only access for the monitors and auditors and anyone else authorized to inspect or verify records.

Following the DCOC monitoring SOPs, the monitors will verify that the site is conducting the clinical trial and generating, documenting (recording), and reporting data in compliance with the protocol, the Site Monitoring Plan, site-specific SOPs, the ICH GCP E6(R2), and applicable regulatory requirements.

The DCOC will implement quality control procedures for the database and DCOC-maintained records in accordance with the Site Monitoring Plan, MOP, data safety monitoring plan (DSMP), and applicable SOPs. The DCOC may communicate information about any data anomalies to the site(s) for clarification/resolution.

The DCOC will address issues uncovered during quality assurance, quality control, or monitoring activities through simple corrections or root-cause analysis, followed by instituting corrective and preventative action (CAPA), as appropriate and as described in the MOP.

##### Data quality assurance

To assure the quality of the data collected, the protocol study team will provide training specific to entering data related to the endpoints of initiation and completion of COVID-19 vaccination. The site research team will re-confirm a subsample of participating caregivers’ report of COVID-19 vaccination for their child/children.

The DCOC will provide sites with the randomly selected caregiver IDs for re-confirmation. The DCOC site manager will meet with the site research coordinator and/or site investigator to review any discrepancies. Together they will discuss and document the corrective action for each error identified. The DCOC will create manual queries in the EDC system to make any necessary corrections to the data. The protocol study team will provide sites that have an error rate above the predefined threshold with additional training, a site-specific assessment of the data collection process, and suggestions for process improvement. The protocol study team will track sites by their error rates. The protocol study team will share practices of those sites with exceptionally low error rates with sites working to improve their own process. The protocol study team will review errors during monthly team calls. If errors exceed the predefined threshold on 2 consecutive reviews, a remediation plan will be requested and shared with the study sponsor.

#### Data handling and record keeping

##### Data collection and management responsibilities

A formal data management plan will describe and document the data and workflow for the trial. The data management plan and associated documentation will specify all operations performed on data from origination to database lock, including detailed descriptions of source documentation, case report forms (CRF), instructions for completing forms, data handling and record keeping procedures, procedures for data monitoring, and reconciliation procedures and coding dictionaries to be used, if applicable. The data management plan will also describe the specific data collection and management responsibilities required of the sponsor, PIs, site investigators, site awardees, clinics, and DCOC. The data management plan contents will be consistent with those described in the Good Clinical Data Management Practices (GCDMP). The DCOC will provide the data management plan components that document operations performed on the data to the PIs for review and approval prior to implementation.

Data collection is the responsibility of the trial staff at the individual sites under the supervision of the site investigator. The site investigator is responsible for ensuring the accuracy, completeness, legibility, and timeliness of the data reported. All source documents must be completed using standard good documentation practices (i.e., the ALCOA-C method [attributable, legible, contemporaneous, original, accurate and complete]).

It is best practice for site coordinators to use hardcopies of any data recorded on paper case report forms or trial visit worksheets/assessment forms as source document worksheets for recording data for each participant consented. Data recorded in EDC derived from source documents must be consistent with the data recorded on the source documents.

Site personnel will enter data (including demographics and intervention-specific questionnaires) into an EDC system that complies with HIPAA regulations, provided by the DCOC. During this process, sites will assign each participant a participant identification number and include this number in the EDC and source documents. The EDC system includes password protection and internal quality checks, such as automatic range checks, to identify data that appear inconsistent, incomplete, or inaccurate. Study personnel will enter clinical data directly from the source documents.

##### Study records retention

Throughout the course of the trial, all sites will retain the source documents on site in accordance with current site-specific medical record storage procedures.

Sites must retain all trial documents in accordance with local and/or federal regulations, whichever is most stringent. Sites will not destroy any records without the written consent of the DCOC. The DCOC will inform all site investigators when they no longer need to retain these documents.

##### Protocol deviations

Protocol deviations are any instances in which study team members and site personnel (e.g., site investigator, site coordinator) do not follow the study procedures as written in the protocol. Protocol deviations are not allowed, unless the DCOC or Operational PI gives specific written permission for the deviation. Anyone who receives written permission for a protocol deviation must keep this with other study documentation. Sites must record all deviations in the trial source documents. Whenever a deviation occurs, the DCOC will conduct an assessment (which they do not need to write) of the severity and risk of the deviation.

Depending on the results of the assessment, the DCOC will request/ensure that there is either a corrective action or a simple one-time correction, as appropriate. A corrective action institutes a process designed to keep the specific problem from reoccurring. Typically, assessors will determine the root cause of the problem to develop the most appropriate corrective action plan.

We will provide the specific methods for handling deviations in the trial-specific MOP and/or trial-specific SOPs.

##### Publications and data sharing policy

We will conduct this trial in accordance with the following publication and data sharing policies and regulations:

- *NIH Public Access Policy*, which ensures that the public has access to the published results of NIH-funded research. It requires scientists to submit final peer-reviewed journal manuscripts that arise from NIH funds to the digital archive PubMed Central upon acceptance for publication.
- *ECHO ISPCTN Publications and Presentations Policy*, which ensures accurate, responsible, and efficient communication of findings from ECHO ISPCTN clinical trials. The ECHO ISPCTN Steering Committee has approved and ratified the ECHO ISPCTN Publications and Presentations Policy, which includes representatives from all site awardees, as well as representatives from the NIH and the DCOC.
- *NIH Data Sharing Policy and the policy on the Dissemination of NIH-Funded Clinical Trial Information and the Clinical Trials Registration and Results Information Submission Rule*. We will register this trial at ClinicalTrials.gov, and we will submit trial results to ClinicalTrials.gov. In addition, we will make every attempt to publish results in peer-reviewed journals. Other researchers my request data from this trial by contacting Jeannette Lee, PhD, at the DCOC.

##### Conflict of interest policy

The independence of this trial from any actual or perceived influence, such as by the pharmaceutical industry, is critical. Therefore, we will disclose and manage any actual conflict of interest of persons who have a role in the design, conduct, analysis, publication, or any aspect of this trial. Furthermore, persons who have a perceived conflict of interest will be required to have such conflicts managed in a way that is appropriate to their participation in the design and conduct of this trial. The trial leadership in conjunction with the NIH ECHO office has established policies and procedures for all trial group members to disclose all conflicts of interest and will establish a mechanism for the management of all reported dualities of interest.

## Discussion

### Operational considerations

#### Participating clinics

A total of 29 clinics from 15 states are participating in this study. Prior experience recruiting participants to complete a qualitative assessment of concerns caregivers have regarding vaccinating children against COVID-19 highlighted the important of local site research team members assisting with participants engaging in study activities organized by the central protocol team. Consequently, local site research coordinators will assist with following up with participants who do not download the app after the central study team sends out instructions for downloading the app and retrieving participants’ registration information. Local enrolling site research staff will also assist in participants accessing technical support resources provided by the central study team as necessary. Both activities pose the risk of unblinding local study staff to participants’ app assignment. 

#### Public health impact

In this study, we are estimating that using the Vaccine Uptake app will result in a 10% increase in the proportion of children who will complete COVID-19 vaccination. When looking at the impact of a 10% increase in pediatric vaccination rates, based on a US population of 53.7 million children 5–17 years of age [58], such an increase would result in an additional 5 million vaccinated children. Previous research has shown that app-based vaccine uptake interventions are less costly than more traditional methods, such as paper handouts and communication tools. We estimate a cost of $10,000 dollars for the first year of a customized version of the Vaccine Uptake app per state. Deployment of the Vaccine Uptake app nationally would cost a little as $500,000 dollars per year, or 10 cents per additional child who completes COVID-19 vaccination.

## Trial status

Protocol version: 07

Protocol date: 21 July 2022

Date of recruitment initiation: 07/18/2022

Estimated recruitment completion date: 01/01/2023

## Data Availability

No specimens will be collected or stored for this trial. Regarding stored data, site investigators, site coordinators, and study team members will document all trial interactions, and these will be password protected in a secured facility/location. The study team will place participant’s de-identified data and other limited information, such as race and ethnic group, into one or more centralized database(s). The study team will share this data in compliance with the NIH data sharing policy. For future studies using any procedures or analysis not specified in this protocol, IRB approval is required. If another investigator/collaborator has a meaningful purpose for accessing the data retrieved in this protocol, the DCOC must consult the PIs and the cIRB must approve.

## Abbreviations

AAP: American Academy of Pediatrics
ACIP: Advisory Committee on Immunization Practices
AE: Adverse event
App: Application
CAPA: Corrective and preventative action
CDC: Center for Disease Control and Prevention
CFR: Code of Federal Regulations
cIRB: Central Institutional Review Board
COVID-19: Coronavirus disease 2019
CRF: Case report form
DCOC: Data Coordinating and Operations Center
DSMB: Data Safety Monitoring Board
DSMP: Data Safety Monitoring Plan
ECHO: Environmental influences on Child Health Outcomes
EDC: Electronic data capture system
EUA: Emergency Use Authorization
FDA: Food and Drug Administration
GCDMP: Good Clinical Data Management Practices
GCP: Good Clinical Practice
HBM: Health Belief Model
HIPAA: Health Insurance Portability and Accountability Act
HPV: Human papillomavirus
ICF: Informed consent form
ICH: International Conference on Harmonisation
IRB: Institutional review board
ISPCTN: IdeA States Pediatric Clinical Trials Network
ITT: Intention-to-treat
mHealth: Mobile Health
MIS-C: Multisystem Inflammatory Syndrome in Children
mITT: Modified ITT
MMR: Measles, mumps, and rubella
MOP: Manual of procedures
NCT: National Clinical Trial
NIH: National Institutes of Health
OHRP: Office for Human Research Protections
PHI: Protected health information
PI: Principal Investigator
PP: Per-protocol
PROS: Pediatric Research in Outpatient Settings
QA: Quality assurance
QC: Quality control
QR: Quick response
RUCA: Rural-urban community area
SAE: Serious adverse event
SAP: Statistical Analysis Plan
SMART IRB: Streamlined, Multisite, Accelerated Resources for Trials IRB
SOA: Schedule of activities
SOP: Standard operating procedure
SUS: System Usability Scale
UAMS: University of Arkansas for Medical Sciences
UP: Unanticipated problem
UPIRTSO: Unanticipated problems involving risks to subjects or others
UNMC: University of Nebraska Medical Center
US: United States

## References

[1] Mahase E (2020). Covid-19: Moderna applies for US and EU approval as vaccine trial reports 94.1% efficacy. BMJ..

[2] Oliver SE, Gargano JW, Marin M (2020). The Advisory Committee on Immunization Practices’ Interim Recommendation for Use of Pfizer-BioNTech COVID-19 Vaccine - United States, December 2020. MMWR Morb Mortal Wkly Rep.

[3] Khubchandani J, Sharma S, Price JH, Wiblishauser MJ, Sharma M, Webb FJ (2021). COVID-19 Vaccination Hesitancy in the United States: A Rapid National Assessment. J Community Health.

[4] COVID Collaborative LR, UnidosUS, NAACP. COVID Collaborative Survey: Coronavirus Vaccination Hesitancy in the Black and Latinx Communities November 23, 2020 2020.

[5] (U.S.) Centers for Disease Control and Prevention. Risk for COVID-19 Infection, Hospitalization, and Death By Race/Ethnicity. https://www.cdc.gov/coronavirus/2019-ncov/covid-data/investigations-discovery/hospitalization-death-by-race-ethnicity.html. Accessed 8 Aug 2020.

[6] Sanchez GaP, JM Skepticism and mistrust challenge COVID vaccine uptake for Latinos. In. How We Rise. Vol 2021: Brookings Institute; 2021. https://www.brookings.edu/blog/how-we-rise/2021/01/25/skepticism-and-mistrust-challenge-covid-vaccine-uptake-for-latinos/. Accessed 12 Mar 2021.

[7] Health Issues and Priorities: Latino Voters in the 2020 Electorate. https://www.unidosus.org/publications/2089-health-issues-and-priorities-latino-voters-in-the-2020-electorate/. Accessed 15 Dec 2020.

[8] Chou WS, Burgdorf CE, Gaysynsky A, Hunter CM (2020). COVID-19 Vaccination Communication: Applying Behavioral and Social Science to Address Vaccine Hesitancy and Foster Vaccine Confidence.

[9] National Institutes of Health Community Engagement Alliance (CEAL), Communication Work Group. Addressing COVID-19 Misinformation: A Tip Sheet for Health Professionals Working with Community Members. Toolkit: Adapting Fact-Based Information to the Needs of Communities: A Guide Web site. https://covid19community.nih.gov/communication-resources. Published 2021. Updated April 19, 2021. Accessed 14 Sept 2021.

[10] Prevention CDC. Different COVID-19 Vaccines. Centers for Disease Control and Prevention. 2020. https://www.cdc.gov/coronavirus/2019-ncov/vaccines/different-vaccines.html. Published 2021. Accessed 1 June 2020.

[11] Centers for Disease Control and Prevention. Weekly Updates by Select Demographic and Geographic Characteristics: Provisional Death Counts for Coronavirus Disease 2019 (COVID-19). https://www.cdc.gov/nchs/nvss/vsrr/covid_weekly/index.htm. Published 2022. Accessed 8 Sept 2022.

[12] Jenco M. Pfizer plans to request COVID-19 vaccine EUA for ages 2–11 in September. American Academy of Pediatrics. https://www.aappublications.org/news/2021/05/04/pfizer-covid-vaccine-children-050421. Published 2021. Accessed 13 May 2021.

[13] Szilagyi PG, Shah MD, Delgado JR, Thomas K, Vizueta N, Cui Y, Vangala S, Shetgiri R, Kapteyn A. Parents’ Intentions and Perceptions About COVID-19 Vaccination for Their Children: Results From a National Survey. Pediatrics. 2021;3:e2021052335. 10.1542/peds.2021-052335 PMC10116994 34344800

[14] Nguyen KH, Srivastav A, Razzaghi H (2021). COVID-19 Vaccination Intent, Perceptions, and Reasons for Not Vaccinating Among Groups Prioritized for Early Vaccination - United States, September and December 2020. MMWR Morb Mortal Wkly Rep.

[15] Snowden J, Darden P, Palumbo P, Saul P, Lee J (2018). The institutional development award states pediatric clinical trials network: building research capacity among the rural and medically underserved. Curr Opin Pediatr.

[16] Saelee R, Zell E, Murthy BP, Castro-Roman P, Fast H, Meng L, Shaw L, Gibbs-Scharf L, Chorba T, Harris LQ, Murthy N (2022). Disparities in COVID-19 Vaccination Coverage Between Urban and Rural Counties - United States, December 14, 2020-January 31, 2022. MMWR Morb Mortal Wkly Rep.

[17] Ndugga N, Pham O, Hill L, Artiga S, Parker N (2022). Latest Data on COVID-19 Vaccinations by Race/Ethnicity.

[18] Champion VSC, Glanz KRB, Viswanath K (2008). The Health Belief Model. Health Behavior and Health Education.

[19] Mercadante AR, Law AV. Will they, or Won’t they? Examining patients’ vaccine intention for flu and COVID-19 using the Health Belief Model. Res Social Adm Pharm. 2020. 10.1016/j.sapharm.2020.12.012 PMC7833824 33431259

[20] Gilkey MB, Magnus BE, Reiter PL, McRee AL, Dempsey AF, Brewer NT. The Vaccination Confidence Scale: a brief measure of parents’ vaccination beliefs. Vaccine. 2014;32(47):6259–65. 10.1016/j.vaccine.2014.09.007 PMC4418546 25258098

[21] The Security Rule. Health Information Privacy Web site. Published 2017. Accessed 12 June 2017.

[22] Keelan KWKAJ (2014). Using Mobile Technology To Overcome Jurisdictional Challenges To A Coordinated Immunization Policy. Health Affairs Blog.

[23] Fadda M, Galimberti E, Fiordelli M, Romanò L, Zanetti A, Schulz PJ (2017). Effectiveness of a smartphone app to increase parents’ knowledge and empowerment in the MMR vaccination decision: A randomized controlled trial. Hum Vaccin Immunother.

[24] Wilson K, Atkinson KM, Westeinde J (2015). Apps for immunization: Leveraging mobile devices to place the individual at the center of care. Hum Vaccin Immunother.

[25] Shourie S, Jackson C, Cheater FM (2013). A cluster randomised controlled trial of a web based decision aid to support parents’ decisions about their child’s Measles Mumps and Rubella (MMR) vaccination. Vaccine.

[26] Tubeuf S, Edlin R, Shourie S, Cheater FM, Bekker H, Jackson C (2014). Cost effectiveness of a web-based decision aid for parents deciding about MMR vaccination: a three-arm cluster randomised controlled trial in primary care. Br J Gen Pract.

[27] Levine G, Salifu A, Mohammed I, Fink G (2021). Mobile nudges and financial incentives to improve coverage of timely neonatal vaccination in rural areas (GEVaP trial): A 3-armed cluster randomized controlled trial in Northern Ghana. PLoS ONE.

[28] Brewer NT (2021). What Works to Increase Vaccination Uptake. Acad Pediatr.

[29] Fu LY, Bonhomme LA, Cooper SC, Joseph JG, Zimet GD (2014). Educational interventions to increase HPV vaccination acceptance: a systematic review. Vaccine.

[30] Larkey LK, Hecht M (2010). A model of effects of narrative as culture-centric health promotion. J Health Commun.

[31] Hopfer S, Clippard JR (2011). College women’s HPV vaccine decision narratives. Qual Health Res.

[32] Wilson K, Atkinson KM, Penney G (2015). Development and release of a national immunization app for Canada (ImmunizeCA). Vaccine.

[33] Feemster KA, Head KJ, Panozzo CA, O’Dell SM, Zimet GD, Kornides ML (2021). Efficacy of tailored messages to improve behavioral intent to accept HPV vaccination among mothers may be moderated by sociodemographics. Prev Med Rep.

[34] Myers A, Ipsen C, Lissau A (2022). COVID-19 vaccination hesitancy among Americans with disabilities aged 18–65: An exploratory analysis. Disabil Health J.

[35] Kerns EK, Staggs VS, Fouquet SD, McCulloh RJ (2019). Estimating the impact of deploying an electronic clinical decision support tool as part of a national practice improvement project. J Am Med Inform Assoc.

[36] Richardson KM, Fouquet SD, Kerns E, McCulloh RJ (2019). Impact of Mobile Device-Based Clinical Decision Support Tool on Guideline Adherence and Mental Workload. Acad Pediatr..

[37] McCulloh RJ, Fouquet SD, Herigon J, Biondi EA, Kennedy B, Kerns E, DePorre A, Markham JL, Chan YR, Nelson K, Newland JG (2018). Development and implementation of a mobile device-based pediatric electronic decision support tool as part of a national practice standardization project. J Am Med Inform Assoc.

[38] Roberts JR, Morella K, Dawley EH (2018). Direct-to-adolescent text messaging for vaccine reminders: What will parents permit?. Vaccine.

[39] Carney PA, Hatch B, Stock I (2019). A stepped-wedge cluster randomized trial designed to improve completion of HPV vaccine series and reduce missed opportunities to vaccinate in rural primary care practices. Implement Sci.

[40] Taylor JA, Darden PM, Brooks DA, Hendricks JW, Wasserman RC, Bocian AB (2002). Association between parents’ preferences and perceptions of barriers to vaccination and the immunization status of their children: a study from Pediatric Research in Office Settings and the National Medical Association. Pediatrics.

[41] Detoc M, Launay O, Dualé C (2019). Barriers and motivations for participation in preventive vaccine clinical trials: Experience of 5 clinical research sites. Vaccine.

[42] Mobile Technology and Home Broadband 2021. Pew Research Center, Washington, D.C. June 2021 https://www.pewresearch.org/internet/2021/06/03/mobile-technology-and-home-broadband-2021. Accessed 21 Apr 2022.

[43] Rural-Urban Commuting Area Codes. https://data.nal.usda.gov/dataset/rural-urban-commuting-area-codes. Published. Accessed 15 Sept 2022

[44] Rural-Urban Commuting Area Codes. US. Department of Agriculture. Updated August 17,2020. https://ers.usda.gov/data-products/rural-urban-commuting-area-codes.aspx. Accessed 30 July 2021

[45] NIH Rapid Acceleration of Diagnostics in Underserved Populations (RADx-UP) Common Data Elements. 2020. https://radx-up.org/wp-content/uploads/2021/01/RADx-UP-_-REDCap20201230_codebook-.pdf. Accessed 1 May 2021.

[46] Hesitancy SWGoV (2014). Report of the Sage Working Group on Vaccine Hesitancy.

[47] Usability Evaluation In Industry. 1 ed. London, UK: CRC Press; 1996.

[48] Center for Climate Change Communication. https://www.climatechangecommunication.org/. Accessed 14 Sept 2021.

[49] Lewandowsky, S, Cook, J, Ecker, U K H, et al. The Debunking Handbook 2020. Available at https://sks.to/db2020. 10.17910/b7.1182.

[50] Southwell BG, Niederdeppe J, Cappella JN (2019). Misinformation as a misunderstood challenge to public health. Am J Prev Med.

[51] Murthy BP, Zell E, Saelee R (2021). COVID-19 Vaccination Coverage Among Adolescents Aged 12–17 Years — United States, December 14, 2020–July 31, 2021. MMWR Morb Mortal Wkly Rep.

[52] Dai H, Saccardo S, Han MA, Roh L, Raja N, Vangala S, Modi H, Pandya S, Sloyan M, Croymans DM (2021). Behavioural nudges increase COVID-19 vaccinations. Nature.

[53] Fadda M, Galimberti E, Fiordelli M, Romanò L, Zanetti A, Schulz PJ. Effectiveness of a smartphone app to increase parents’ knowledge and empowerment in the MMR vaccination decision: A randomized controlled trial. Hum Vaccin Immunother. 2017;13(11):2512–21. 10.1080/21645515.2017.1360456 PMC5703372 29125783

[54] Shourie S, Jackson C, Cheater FM, Bekker HL, Edlin R, Tubeuf S, Harrison W, McAleese E, Schweiger M, Bleasby B, Hammond L. A cluster randomised controlled trial of a web based decision aid to support parents’ decisions about their child’s Measles Mumps and Rubella (MMR) vaccination. Vaccine. 2013;31(50):6003–10. 10.1016/j.vaccine.2013.10.025 PMC3898271 24148574

[55] Kempe A, Stockwell MS, Szilagyi P (2021). The Contribution of Reminder-Recall to Vaccine Delivery Efforts: A Narrative Review. Acad Pediatr.

[56] de Cock C, van Velthoven M, Milne-Ives M, Mooney M, Meinert E. Use of Apps to Promote Childhood Vaccination: Systematic Review. JMIR Mhealth Uhealth. 2020;8(5):e17371. 10.2196/17371 PMC7265109 32421684

[57] The HIPAA Privacy Rule. Health Information Privacy Web site. https://www.hhs.gov/hipaa/for-professionals/privacy/index.html. Accessed 13 Mar 2021.

[58] Kids Count Data Center. Child population by age group in the United States. https://datacenter.kidscount.org/data/tables/101-child-population-by-age-group#detailed/1/any/false/574,1729,37,871,870,573,869,36,868,867/62,63,64,6,4693/419,420. Accessed 15 Sept 2021.

