# Correction: Improving pediatric COVID-19 vaccine uptake using an mHealth tool (MoVeUp): study protocol for a randomized, controlled trial

**DOI:** 10.1186/s13063-024-07951-y

**Published:** 2024-02-08

**Authors:** Russell J. McCulloh, Paul M. Darden, Jessica Snowden, Songthip Ounpraseuth, Jeannette Lee, Martina Clarke, Sophia R. Newcomer, Linda Fu, DeAnn Hubberd, Jaime Baldner, Maryam Garza, Ellen Kerns

**Affiliations:** 1 Children’s Hospital & Medical Center, 8200 Dodge St, Omaha, NE 68114 USA; 2 University of Arkansas for Medical Sciences, 4301 West Markham, Little Rock, AR 72205 USA; 3 College of Information Science & Technology, University of Nebraska Omaha, 172 Peter Kiewit Institute, 1110 South 67Th Street, Omaha, NE 68182 USA; 4 School of Public Health and Community Health Sciences, University of Montana, Skaggs Building Room 177, 32 Campus Drive, Missoula, MT 59812 USA; 5 Ofce of the Director, National Institutes of Health, 11601 Landsdown Sreet, Rockville, MD 20852 USA; 6 University of Nebraska Medical Center, 42Nd and Emile St, Omaha, NE 68131 USA

**Correction: Trials 23, 911 (2022)**

**https://doi.org/10.1186/s13063-022-06819-3**

Following publication of the original article [1], we have been notified that the Competing section needs to be updated.

Originally published Competing interests section:

“The authors declare that they have no competing interests.”

Corrected Competing interests section:

“Dr J Snowden is currently a consultant with Pfizer. The authors confirm, however, that at the time of writing this paper, Dr Snowden did not engage in any commercial or promotional activity of any specific product. The protocol was written, revised, and approved prior to Dr. Snowden's engagement with Pfizer, and had no knowledge of this protocol's existence prior to its publication.”

The original article has been corrected.
